# Extracellular Vesicles Secreted by Cancer‐Associated Fibroblasts Drive Non‐Invasive Cancer Cell Progression to Metastasis via TGF‐β Signalling Hyperactivation

**DOI:** 10.1002/jev2.70055

**Published:** 2025-03-16

**Authors:** Adilson Fonseca Teixeira, Yanhong Wang, Josephine Iaria, Peter ten Dijke, Hong‐Jian Zhu

**Affiliations:** ^1^ Department of Surgery (The Royal Melbourne Hospital) The University of Melbourne Parkville Victoria Australia; ^2^ Huagene Institute, Kecheng Science and Technology Park Nanjing Jiangsu China; ^3^ Department of Cell and Chemical Biology, Oncode Institute Leiden University Medical Center Leiden The Netherlands

**Keywords:** cancer‐associated fibroblasts, circulating tumour cells, extracellular vesicles, metastasis, TGF‐β, therapy, tumour microenvironment

## Abstract

Metastasis is the leading cause of cancer‐related deaths. Cancer‐associated fibroblasts (CAFs) are abundant components within the tumour microenvironment, playing critical roles in metastasis. Although increasing evidence supports a role for small extracellular vesicles (sEVs) in this process, their precise contribution and molecular mechanisms remain unclear, compromising the development of antimetastatic therapies. Here, we establish that CAF‐sEVs drive metastasis by mediating CAF‐cancer cell interaction and hyperactivating TGF‐β signalling in tumour cells. Metastasis is abolished by genetically targeting CAF‐sEV secretion and consequent reduction of TGF‐β signalling in cancer cells. Pharmacological treatment with dimethyl amiloride (DMA) decreases CAFs’ sEV secretion, reduces TGF‐β signalling levels in tumour cells and abrogates metastasis and tumour self‐seeding. This work defines a new mechanism required by CAFs to drive cancer progression, supporting the therapeutic targeting of EV trafficking to disable the driving forces of metastasis.

## 1 Introduction

Despite improvements in breast cancer patient survival, metastasis is still the main cause of breast cancer‐related deaths (Dillekas et al. 2019; Sung et al. 2021; Waks and Winer 2019). Increasing evidence indicates that non‐cancer cells within the tumour microenvironment (TME) play a critical role in cancer progression and may determine whether cancer cells metastasize (Teixeiraet al. 2022; de Visser and Joyce 2023). Cancer‐associated fibroblasts (CAFs) are the most abundant cells in the TME of breast cancers, playing important roles in cancer cell invasion and metastasis (Sahai et al. 2020; Liu et al. 2019; Shoucair et al. 2020). Although different mechanisms have been proposed to explain how CAFs drive cancer cell invasion, their precise contribution still requires further investigation as current knowledge gaps compromise the development of efficient anticancer therapies.

Extracellular vesicles (EVs) have gained increased attention as major mechanisms in CAF‐driven cancer progression (Shoucair et al. 2020; Li et al. 2021). EVs are nanosized particles that mediate cell‐cell communication by transporting nucleic acids, lipids, carbohydrates and proteins (Greening et al. 2015; Raposo and Stahl 2019; van Niel et al. 2018; Xu et al. 2016). Interestingly, we have recently reported that highly invasive breast cancer cells rely on small EVs for the autocrine hyperactivation of the TGF‐β signalling that is required for metastasis (Teixeira et al. 2023). TGF‐β signalling is initiated by ligand‐receptor binding, which induces transphosphorylation of TGF‐β receptor type I (TβRI) by TGF‐β receptor type I (TβRII) (Heldin and Moustakas 2016). If not inhibited by SMAD7, activated TβRI phosphorylates and activates the intracellular effectors SMAD2/3, promoting their association with SMAD4, and accumulation into the nucleus, where this SMAD complex controls the expression of selective genes (Macías‐Silva et al. 1996; Zhang et al. 1996; Nakao et al. 1997). Importantly, several TGF‐β signalling inhibitors exist, acting on‐target and blocking cancer progression in vitro and in vivo. Yet, none of the TGF‐β signalling inhibitors evaluated in cancer clinical trials have so far succeeded in prolonging patient survival (Fonseca Teixeira et al. 2023; Teixeira et al. 2020; Wu et al. 2023). Thus, discovering more precise mechanisms regulating TGF‐β signalling is needed for the development of efficient anti‐metastatic therapies.

In this study, we use breast cancer models and establish that small EVs secreted by CAFs drive metastasis by unlocking the invasive potential of otherwise non‐invasive cancer cells. Mechanistically, we show that CAF‐sEVs not only activate, but rather hyperactivate the TGF‐β signalling in breast cancer cells in vitro and in vivo. Consequently, genetic and pharmacologic inhibition of sEV trafficking efficiently inhibited CAF‐induced breast cancer metastasis to multiple organs and tumour self‐seeding. Thus, we demonstrate a novel therapeutic strategy that targets CAF‐sEV trafficking within the TME to prevent cancer progression and metastasis.

## 2 Materials and Methods

### 2.1 Cell Lines and Cell Cultures

Human MCF7 and MDA‐MB‐231 (MDA231) breast cancer cell lines were purchased from American Type Culture Collection (ATCC). Modified breast cancer cell lines (MDA.Gluc and MCF7.Gluc) were labelled with Gaussia luciferase by transfection with Gaussian‐luc cDNA construct. The constitutive expression of Gaussia luciferase was confirmed by luciferase assay in cells resistant to neomycin after clone selection. Human breast CAFs used in this study refer to 19TT cells that were previously established and kindly provided by Professor John W. M. Martens (Martens et al. 2003). 19TT CAFs were originally immortalized by transduction with retroviral vector containing the human telomerase reverse transcriptase (hTERT) and have been previously used in independent studies (Martens et al. 2003; Ren et al. 2019; Dittmer and Dittmer 2022; Dittmer and Dittmer 2018; Dittmer et al. 2011; Leyh et al. 2015). Stable Rab27a knockdown was established in 19TT CAFs (19TT.Rab27a.shRNA) infection with Rab27a shRNA lentivirus (Santa Cruz Biotechnology, sc‐41834‐v) using Polybrene (sc‐134220, Santa Cruz Biotechnology). Efficient knockdown was confirmed by western blot in cells resistant to high puromycin concentration (> 50 µg/mL). Cell lines were cultured in Dulbecco's modified Eagle's medium (DMEM) (Gibco, Thermo Fisher Scientific) supplemented with 10 µg/mL penicillin and 100 µg/mL streptomycin (Invitrogen, Thermo Fisher Scientific) and 10% foetal calf serum (FCS) (HyCloneTM, GE Healthcare Life Sciences). Breast cancer cell lines and CAFs were cultured at 37°C with 10% CO_2_ in a humidified atmosphere.

### 2.2 DNA Constructs and Adenovirus Production

**A**denoviruses used in this study (Ad‐CAGA‐FLuc, Ad‐CMV‐GLuc, Ad‐APRE‐Fluc, Ad‐BRE‐FLuc, Ad‐TCF‐FLuc, Ad‐CMV‐GFP, Ad‐CMV‐TdTom and Ad‐CMV‐Flag‐SMAD7) were generated and amplified as described in previous studies (Luwor et al. 2015; Luwor et al. 2011). TGF‐β/SMAD3 signalling activity was quantified by luciferase assay using by initially cloning *pCAGA‐FLuc* DNA into a *pENTR 1A* entry clone vector (Invitrogen, Thermo Fisher Scientific), obtaining *pENTR‐CAGA‐FLuc*. After, a recombination system (attL‐arrR) was used with a *pAd/PL‐DEST* Destination vector (Invitrogen, Thermo Fisher Scientific), obtaining the adenovirus plasmid *pAdCAGA_12_‐Luc*. Plasmid was transfected into 293A cells to produce adenovirus following digestion with *Pac I*. Lipofectamine LTX transfection reagent (Invitrogen, Thermo Fisher Scientific) was used for plasmid transfection. Cell lysis was monitored, and floating cells were harvested for the amplification and determination of adenovirus titre. Other adenovirus used in this work (Ad‐CMV‐GLuc, Ad‐APRE‐Fluc, Ad‐BRE‐FLuc, Ad‐TCF‐FLuc, Ad‐CMV‐GFP, Ad‐CMV‐TdTom and Ad‐CMV‐Flag‐SMAD7) were similarly produced.

### 2.3 Small Molecule Inhibitors and Cell Treatment

Recombinant human (rh)TGF‐β1 (100‐21, PeproTech), rhBMP4 (120‐05, PeproTech), and rhIL‐6 (200‐06, PeproTech) were used as positive controls to treat cell cultures at indicated concentrations and time intervals. Large (l)EVs, small (s)EVs, and cell culture conditioned medium depleted in EVs were also used to treat cell cultures and cell response was evaluated regarding activation of molecular pathways and functional effects. EV treatment was normalized by total protein content or TGF‐β activity as indicated. The small molecule SB431542 (S4317, Sigma–Aldrich) was used as TGF‐β signalling inhibitor based on its confirmed specificity for the kinase activity of TGF‐β type 1 receptor. Ad‐CMV‐Flag‐SMAD7 adenovirus (home‐made) was also used as TGF‐β signalling inhibitor by promoting SMAD7 overexpression in infected cell cultures. 5‐(N,N‐Dimethyl)amiloride hydrochloride (A4562, Sigma–Aldrich), heparin (H3393, Sigma–Aldrich) or 4‐Nitrophenyl β‐D‐xylopyranoside (PNP‐Xyl) (N2132, Sigma–Aldrich) were used based on reported activity on EV trafficking inhibition.

### 2.4 Isolation and Characterization of EVs

EVs were investigated according to Minimal information for studies of EVs (Welsh et al. 2024). EVs secreted by 19TT CAFs were isolated from cell culture conditioned medium by submitting samples to differential centrifugation and filtration (Figure S1A). Briefly, 19TT CAFs were seeded and cultured in Petri dishes (150 mm × 25 mm) until approximately 70%–80% cell culture confluency was achieved. Cell culture medium was then discarded, and fresh serum‐free medium (SFM) added to cell cultures to avoid contamination by EV‐containing FCS. Cells were incubated in SFM ± recombinant human (rh)TGF‐β1 for 2–4 h following the procedure previously described by our group to increase the TGF‐β activity in collected EVs while also mimicking physiological availability of TGF‐β in the environment of breast cancers (Teixeira et al. 2023). Next, conditioned medium was collected in 50 mL tubes and centrifuged (300 × *g*, 5 min, 4°C) aiming at removing floating cells and cell debris. Sequential centrifugation was then used to remove additional debris (2000 × *g*, 15 min, 4°C followed by 3166 × *g*,15 min, 4°C). The generated pellet was discarded and resulting supernatant (solution 1) was centrifuged (3166 × *g*, 4°C) for concentration in 100K NMWL tubes (Amicon Ultra‐15, Merck Millipore). Filters in concentration tube were then washed twice with ice‐cold SFM or phosphate‐buffered saline (PBS) for the removal of residual soluble factors. About 200 µL of concentrated solution (solution 2) was then centrifuged (10,000 × g, 90 min, 4°C). Large EVs (10K pellet) were resuspended in SFM or PBS and stored (4°C). Additionally, supernatant (solution 3) was ultracentrifuged in polycarbonate thick wall tubes (116,000 × g, 20 h, 4°C) for the isolation of small EVs (100K pellet). Pellets containing small vesicles were resuspended in SFM or PBS and stored (4°C) for further analysis. Conditioned medium depleted in EVs (100K supernatant) generated after ultracentrifugation was also collected and stored (4°C) for further analyses. Characterization of isolated EVs was done according to physicochemical properties by evaluating particle morphology, size, and protein content.

Cryo‐electron microscopy (cryo‐EM) was used to analyse 19TT‐EV's morphology whether EVs were resuspended in PBS and stained on formvar carbon‐coated nickel grids with 2% uranyl acetate solution. Tecnai F30 electron microscope equipped with a HAADF STEM detector, a Gatan quantum 965 energy filter, and an upper CETA 4 × 4k CMOS camera were used for the analysis. Additionally, bicinchoninic acid (BCA) protein assay was used for the quantification of EV's total protein by mixing 200 µL Pierce BCA Protein Assay Kit (23225, Thermo Fisher Scientific) with 1–5 µL EV samples. Resulting alterations in solution absorbance were quantified at 562 nm according to the manufacturer's instructions following incubation for 30 min at 37°C. A standard curve using bovine serum albumin (BSA) at different concentrations was used as control. Further, a NanoSight N300 (Malvern Panalytical) coupled with a 488 nm blue laser was used to establish the particle size distribution and particle concentration of solutions containing isolated 19TT‐EVs. Samples were diluted in PBS (viscosity 0.925–0.929 cP) and tested until manufacture's recommended concentrations were obtained. Samples were automatically injected by a syringe pump controlled with a pre‐defined script system. SCMOS camera was used to record particle movement (3–5 videos; 30 s/video; 25 frames/second) following threshold and automatic blur size setting. Nanoparticle tracking analysis software (NTA 3.2 Dev Build 3.2.16) was used for video analysis and to determine mean, mode, median size, and concentration of particles for each sample. Samples were compared by using the same post‐capture parameters.

### 2.5 Luciferase Reporter Assay

Quantification of TGF‐β/SMAD3 signalling pathway activity was done by infecting cells with Ad‐CAGA‐FLuc (MOI: 2000) adenovirus in 96‐well plates overnight. Ad‐CMV‐Gluc (MOI: 200) was used as control. Approximately 24 h after infection and seeding, cell cultures were stimulated as indicated in the result sections. Most experiments included cell culture treatment with recombinant human (rh)TGF‐β1 or 19TT CAF‐secreted EVs at indicated concentrations and time points. Three technical replicates were used per condition in each experiment. Cell culture medium was then aspirated and incubated with 50 µL/well Cell Culture Lyses Reagent (Promega). After lysis (30 min; 4°C), 30 µL/well cell lysates were transferred to a 96‐well opaque reading plate and a GloMax 96 Microplate Luminometer used to quantify the luciferase activity by automated injection of Luciferase Assay Kit (Promega). Response to rhTGF‐β1 or 19TT‐EVs was normalized relative to untreated cell cultures and represented as fold‐change. Similar procedures were used to quantify the TGF‐β/SMAD3 signalling reporter (Ad‐CAGA‐Gluc) activity, BMP/SMAD1/5 signalling reporter (Ad‐BRE‐Fluc) activity, IL‐6/STAT3 signalling reporter (Ad‐APRE‐Fluc) activity, and WNT/TCF signalling reporter (Ad‐TCF‐Fluc) activity.

### 2.6 Determining TGF‐β Activity in Isolated EVs

The concentration of active TGF‐b in EVs was established by dual luciferase assay using MDA‐MB‐231 cells infected with Ad‐CAGA‐FLuc and Ad‐CMV‐GLuc adenoviruses as previously described (Teixeira et al. 2022). Briefly, TGF‐β/SMAD3 signalling reporter (Ad‐CAGA‐FLuc) activity was quantified in cells treated with 19TT CAF‐EVs and 19TT CAF conditioned medium depleted in EVs. Solutions containing EVs or depleted in EVs were used at increasing concentrations as indicated. Obtained results were then interpolated in a standard curve generated by treating cell cultures with rhTGF‐β1 at increasing concentrations. Luciferase activity in solutions with unknown TGF‐β activity (EVs and EV‐depleted conditioned medium) and TGF‐β standard curves were simultaneously quantified to avoid inter‐experiment variation.

### 2.7 Western Blotting

Cell cultures were treated with recombinant human (rh)TGF‐β1 or 19TT CAF‐secreted EVs using concentrations and intervals indicated in the results section. Cell culture medium was aspirated and cell lysis was done by incubating cell cultures with ice‐cold lyses buffer (30 mM HEPES, 1% TritonX‐100, 2 mM MgCl_2_, 150 mM NaCl, 5 mM EDTA, complete protease inhibitor tablet and phosphostop phosphatase cocktail tablet). After cell lysis (30 min, 4°C), lysates transferred to microtubes were cleaned from cell debris by centrifugation at 13,000 rpm (15 min, 4°C). Supernatants were then transferred to new microtubes, and pellets were discarded. Total protein concentration in cell lysates was established by BCA protein assay. Next, samples were mixed with Laemmli sample buffer (4×) (Bio‐Rad laboratories) and heated (10 min, 95°C), following protein separation using Bolt 4%–12%, Bis‐Tris, 1.0 mm, Mini Protein Gels (Thermo Fisher Scientific). Semi‐dry transference was employed by using a nitrocellulose membrane and an iBlot 2 Dry Blotting System (Invitrogen, Thermo Fisher Scientific), followed by blocking though nitrocellulose membrane incubation in 5% skim milk solution (1 h, RT). Membranes were then washed (30 min, RT) and incubated with solution containing primary antibody (4°C, overnight) raised against specific target proteins. After harvesting primary antibody solution, nitrocellulose membranes were washed (30 min, RT) and incubated with secondary antibody conjugated to horse radish peroxidase (HPR) (1 h, RT). Western Lightning ECL Pro Enhanced Chemiluminescence Substrate (PerkinElmer) was used for developing and membranes then were imaged in a ChemiDoc MP Imaging System (Bio‐Rad laboratories) coupled to CCD camera. Total SMAD2 levels and/or β‐Actin levels were used as loading controls. Primary antibodies used in this study were raised against: phospho‐SMAD2 (pSMAD2, home‐made), SMAD2 (610843, BD Biosciences), ZO‐1 (610967, BD Biosciences), E‐cadherin (610182, BD Biosciences), Rab27a (Ab55667, Abcam), Flag M2 (F3165, Sigma–Aldrich), b‐actin (A5441, Sigma–Aldrich), Alix (2171S, Cell Signalling Technology), TSG101 (612696, BD Biosciences) and CD63 (sc‐5275, Santa Cruz Biotechnology). Secondary antibodies used in this study included: Goat anti‐rabbit IgG (H+L)‐HRP conjugate (1706515, Bio‐Rad Laboratories) and goat anti‐mouse (H+L)‐HRP conjugate (1706516, Bio‐Rad Laboratories).

### 2.8 Immunofluorescence Staining

Cell cultures were treated with recombinant human (rh)TGF‐β1 or 19TT CAF‐secreted EVs using concentrations and time intervals indicated in the result section. Cell culture medium was then aspirated, and cells were fixed in a 3.7% formaldehyde solution (5 min, RT), followed by PBS‐washing, Triton‐X‐100 (0.1%) permeabilization (5 min, RT) and PBS‐washing. After blocking with 5% BSA solution (1 h, RT), cells were incubated with primary antibody (1 h, RT). Primary antibody solution was then collected, cells were PBS‐washed, and incubated with secondary antibody conjugated to Alexa Fluor 546 (1 h, RT). Secondary antibody solution was then discarded, cells were PBS‐washed and cell nuclei were stained using Hoechst 33342 (10 min, RT). Nuclei staining solution was aspirated, cells were washed with PBS (once) and double‐distilled water (twice), and emitted fluorescence was visualized in a fluorescent microscope coupled to a CCD camera using magnification lenses indicated in the result section. Primary antibodies used in this study were raised against: ZO‐1 (610967, BD Biosciences) and E‐cadherin (610182, BD Biosciences). The secondary antibodies used in this study was the Goat anti‐mouse IgG (H+L) Alexa Fluor 546 (A‐11003, Thermo Fisher Scientific).

### 2.9 In Vitro Quantification of Cell Numbers

Cell numbers were determined in vitro by quantifying the Gaussia luciferase activity in cells stably or transiently labelled with Gaussia luciferase. Gaussia luciferase‐labelled cells were titred and the luciferase activity was analysed by luciferase assay. Cell lysates were used for this purpose as described in the section ‘Luciferase reporter assay’. Cell numbers and obtained results were then correlated.

### 2.10 Wound Healing Assay

Cells were grown until cell cultures reached 80%–90% confluency and a scratch was done by using a P200 pipette tip aiming at establishing a wound (area without cells) in the monolayer. After aspirating the cell culture medium, cell cultures were PBS‐washed and fresh culture medium was added. Additionally, cell cultures were treated with recombinant human (rh)TGF‐b1, 19TT CAF‐secreted EVs, and small molecule inhibitors as indicated in the result section. Cell culture treatment was done after scratching and wounds were immediately imaged as to establish the initial area of the scratch (0 h). Wound healing was then monitored, and cell cultures were imaged again at the end of the experiment (24 h). A microscope coupled to a CCD camera was used for cell imaging (4× magnification). To evaluate cell migration, cells were cultured in reduced serum (1% FCS) from scratching until the end of the experiment. ImageJ software was used to quantify the wound area at 0 and 24 h after scratching. Cell migration was established at the end of the experiment (24 h) and normalized relative to the area quantified immediately after scratch and treatment (0 h).

Alternatively, fluorescently‐labelled cells (infected with Ad‐CMV‐GFP or Ad‐CMV‐TdTom adenoviruses) were cultured ± 19TT CAFs and cell cultures were scratched as described. After treatment ± rhTGF‐b1 and ± small molecule inhibitors, cell cultures were photographed (0 h) by using a CCD camera coupled to a fluorescence microscope (4x magnification). Cell cultures were monitored and photographed again at the end of the experiment (24 h). The wound area in mono‐ or co‐cultures was quantified by using ImageJ software and cell migration was determined by considering the relative wound closure. Migration of cancer cells was distinguished from CAF migration by exclusively considering the area covered by GFP‐ (or TdTom‐)expressing cells. Bright‐field images showing both cancer cells and CAFs were also acquired as controls.

### 2.11 Quantifying Cell Migration and Cell Invasion in Transwell Inserts

Cancer cells were seeded on top of transwell polycarbonate inserts (8.0 µm pore size, Corning) and treated ± recombinant human (rh)TGF‐b1 or 19TT CAF‐secreted EVs. After treatment, cells were incubated in reduced serum (1% FCS) for 24 h for the evaluation of cell migration. Next, cell culture medium was discarded and remaining cells still on top of the membrane of transwell inserts were removed by using a cotton bud dipped in PBS. Migrated cells located at the bottom of the membrane of transwell inserts were then fixed by incubation in 3.7% formaldehyde solution (5 min, RT). Fixing solution was aspirated and transwell inserted were PBS‐washed. Cell nuclei were stained by incubation in Hoechst 33342 solution (10 min, RT). Following PBS‐washing, cell nuclei were imaged and counted by using a fluorescence microscope coupled to CCD camera (10–20× magnification). Cell invasion was similarly evaluated by staining and counting the number of nuclei fixed at the bottom of the membrane in transwell inserts following seeding in Matrigel‐coated transwell inserts, treatment, and incubation for 48 h.

Alternatively, Gaussia luciferase‐labelled cancer cells were seeded in transwell inserts ± 19TT CAFs and treated with rhTGF‐β1. Cell culture medium was removed 24–48 h post‐treatment and transwell inserts were PBS‐washed. The membranes of transwell inserts were then removed with a scalpel blade, transferred to empty wells, and incubated with 100 µL/well Cell Culture Lyses Reagent (Promega) (30 min, 4°C). Cell lysates (30 µL/well) were transferred to a 96‐well opaque reading plate in duplicates. GloMax 96 Microplate Luminometer and Luciferase Assay Kit (Promega) were then used to quantify the Gaussia luciferase activity specifically expressed by cancer cells. Cancer cell migration was normalized relative to untreated cell cultures and represented as fold‐change. Cell invasion was similarly quantified by adjusting the protocol to use Matrigel‐coated transwell inserts and 48 h treatment.

### 2.12 In Vivo Quantification of TGF‐β/SMAD3 Signalling Activity in Breast Cancer Cells

Female NOD‐SCID mice (6‐week‐old) were purchased from Animal Resource Centre (ARC, West Australia). Animals used in this study are characterized by severe combined immune deficiency spontaneous mutation associated with homozygotic mutation in DNA dependent protein kinase active subunit Prkdc^SCID^. Only female mice were used in this study as to evaluate the biology of human female breast cancers. NOD‐SCID mice were orthotopically and contralaterally implanted with unlabelled (wild type) cancer cells and Gaussia luciferase‐labelled cancer cells ± 19TT CAFs. The number of cancer cells and non‐cancer cells injected per mammary fat pad is indicated in the result section. Mice were randomized after tumour implantation and treated as indicated in the result section. Analysis of obtained results was not blind. To establish the impact of isolated EVs, small (s)EVs secreted by 19TT CAFs were isolated, resuspended in PBS, and intratumourally injected. Twenty microliters solution containing 10 µg 19TT‐sEVs (2 ng TGF‐β activity) or vehicle (PBS) were administered per injection. Alternatively, mice were treated with 20 mg/kg dimethyl amiloride (DMA) via intraperitoneal (i.p.) injections. TGF‐β/SMAD3 signalling reporter (Ad‐CGA‐Fluc) activity was quantified in cancer cells at primary tumours (Teixeira et al. 2022; Chen et al. 2018). In Vivo Imaging System (IVIS 200 Series, Caliper Life Sciences) was used to quantify the bioluminescence signal emitted by Firefly luciferase‐labelled cancer cells (Teixeira et al. 2022; Chen et al. 2018). After i.p. injection with 150 mg/kg VivoGlo Luciferin In Vivo Grade (P1043, Promega), animals lying supine were imaged for 30 min at following 1–2 min interval. As control, mice were tilted after a plateau in the emitted bioluminescence was observed (typically 15 min after D‐luciferin injection) and different areas of each tumour were imaged aiming at determining the maximum TGF‐β/SMAD3 signalling activity. Living Image software (V3.2, Caliper Life Sciences) was used to analyse bioluminescence intensities according to the total flux (photons/second) detected in the regions of interest (ROI). Bioluminescence was automatically normalized to background signal as per software default.

### 2.13 Ex Vivo Detection of Circulating Tumour Cells, Metastasis, and Tumour Self‐Seeding

NOD‐SCID mice implanted with Gaussia luciferase‐labelled breast cancer cells ±10TT CAFs were monitored regarding weight and tumour growth. Tumour's largest dimension (d_a_) and smallest dimension (d_b_) obtained by caliper measurements were used in the following equation to determine tumour volume: ($d_{a}\;\times \;d_{b}^{2})/$2. As indicated in the results section, tumours and tissues/organs were harvested after euthanasia for further analyses. Detection of circulating tumour cells (CTCs), metastasis, and tumour self‐seeding was done by ex vivo luciferase assay. Gaussia luciferase activity was associated with the presence of Gaussia luciferase‐labelled cancer cells in harvested samples (Ren et al. 2020). Blood (5 µL/sample; five samples/mouse), lung (5 mg/sample; five samples/mouse), liver (5 mg/sample; five samples/mouse), brain (5 mg/sample; five samples/mouse) and bone (5 mg/sample; five samples/mouse) samples were analysed after lysis. Similarly, tumour self‐seeding (Kim et al. 2009) was assessed unlabelled tumours (5 mg/sample; five samples/tumour/mouse) (Ren et al. 2020). Additionally, two tumour‐naïve NOD‐SCID (non‐implanted animals) were used in specific experiments as negative controls for the detection of Gaussia luciferase‐labelled cells in the blood, peripherical organs, and mammary fat pads.

### 2.14 Statistics

Unless otherwise indicated, in vitro‐derived results are represented as mean ± standard deviation (SD). In vivo‐ and ex vivo‐derived results are represented as mean ± standard error (SEM). Kolmogorov–Smirnov test was used to analyse data distribution. Unpaired Student's *t*‐test and analysis of variance (ANOVA) were adopted for the analysis of results with parametric distribution. Additionally, non‐parametric analyses were used to compare groups with non‐gaussian distribution. Post‐tests were used following ANOVA (and non‐parametric equivalents). All analyses in this study were done by selecting two‐sided tests. Statistically significant differences were considered if *p* < 0.05. InStatGraphpad software (GraphPadInStat version 6.0, GraphPad Prism Software) was used for all statistical analyses reported here.

## 3 Results

### 3.1 CAF‐sEVs Transport TGF‐β Signalling Components and Activate the TGF‐β Signalling in Breast Cancer Cells In Vitro

**FIGURE 1:**
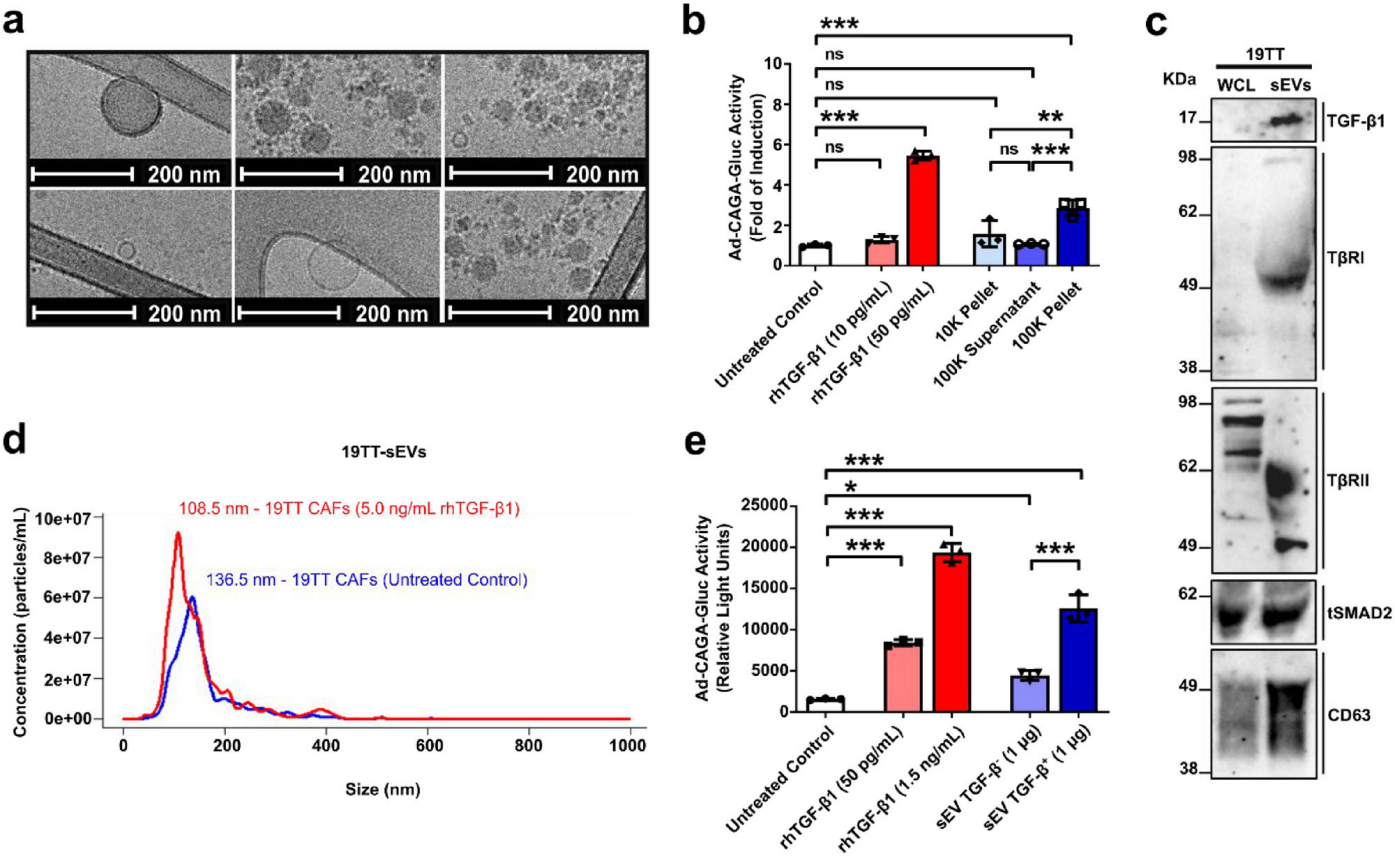
**CAF‐sEVs transport TGF‐β** signalling **components and activate the TGF‐β** signalling **in breast cancer cells in vitro. (a)** 19TT‐sEV morphology was analysed by cryogenic electron microscopy. **(b)** TGF‐β/SMAD signalling reporter (Ad‐CAGA‐Gluc) activity was quantified in MDA231 cells treated for 24 h ± 19TT‐large EVs (10K pellet), EV‐depleted conditioned medium (100K supernatant), or small EVs (100K pellet). Fractions corresponding to 10K pellet and 100K pellet were resuspended in 100 µL serum‐free medium (SFM) during isolation and before use in this analysis. Five microliters per well 10K pellet, 100K supernatant, and 100K pellet were used in this analysis. Treatment with rhTGF‐β1 was used as positive control. **(c)** 19TT‐sEV protein extracts were tested for the expression of extracellular vesicle markers and TGF‐β signalling components by western blot. Whole cell lysates (WCL) were used as positive control. Twenty micrograms of protein extracts were loaded to each lane. **(d)** Nanoparticle tracking analysis (NTA) was used to determine the particle size distribution of sEVs secreted by 19TT CAFs treated ± 5 ng/mL rhTGF‐β1 for 2 h. **(e)** Luciferase assay was used to quantify the TGF‐β/SMAD signalling reporter (Ad‐CAGA‐Gluc) activity in MDA231 cells as in (b). Cells were treated ± 19TT‐sEVs (1 µg/well total protein) isolated from 19TT cells treated ± 5 ng/mL rhTGF‐β1 for 2 h. Results represent mean ± SD of at least three independent experiments (*n* = 3). One‐way ANOVA test followed by Dunn's Multiple Comparison test were used to analyse data in **(b & e)**. ns: statistically non‐significant, *p < 0.05, **p < 0.01, ***p < 0.001.

To investigate the role played by CAF‐EVs in cancer progression, we first isolated and characterized EVs secreted by human breast CAFs (Figure S1a). Immortalized human breast 19TT CAFs were used in our analyses considering typical limitations of primary cells (e.g., slow proliferation and limited lifespan). As analysed by cryogenic electron microscopy, CAF‐EVs isolated from 19TT‐conditioned medium (CM) exhibited typical morphology (Figure 1a). Further, compared with EV‐depleted CM and large EVs, cell cultures treated with small sEVs secreted by CAFs exhibited significantly higher TGF‐β signalling activity (Figure 1b). Interestingly, western blot analysis showed that CAF‐sEVs were not only enriched in the EV marker CD63, but also expressed TGF‐β1, TβRI, TβRII, and SMAD2 (Figure 1c).

Next, CAFs were treated with recombinant human (rh)TGF‐β1 for 2 h and the sEVs secreted by rhTGF‐β1‐treated CAFs were compared with those secreted by untreated cells. This short‐term TGF‐β‐treatment did not significantly change the CAF‐sEV size (Figure 1d) or their total protein content (Figure S1b) but increased the number of sEVs secreted per CAF (Figure S1c). Importantly, compared with sEVs secreted by untreated CAFs (sEV TGF‐β^−^), cell cultures treated with sEVs secreted by TGF‐β‐treated CAFs (sEV TGF‐β^+^) showed increased TGF‐β signalling activity (Figures 1e and S1d,e). Corroborating this result, while sEV TGF‐β^−^ induced breast cancer cell migration, this effect was further enhanced in breast cancer cells treated with sEV TGF‐β^+^ (Figure S1f). Notably, sEV TGF‐β^+^ did not significantly affect the closely related bone morphogenetic protein (BMP)‐SMAD1/5 signalling in breast cancer cells (Figure S1g), nor did it impact the interleukin 6 (IL‐6)/signal transducer and activator of transcription 3 (STAT3) signalling (Figure S1h). Otherwise, CAF‐sEVs activated the Wnt/TCF signalling in breast cancer cells (Figure S1i). This observation agrees with previous findings (Luga et al. 2012), although in the model tested here, the Wnt signalling was activated to a much lesser extent than the TGF‐β signalling.

### 3.2 CAF‐sEVs Hyperactivate the TGF‐β Signalling in Breast Cancer Cells In Vitro

**FIGURE 2:**
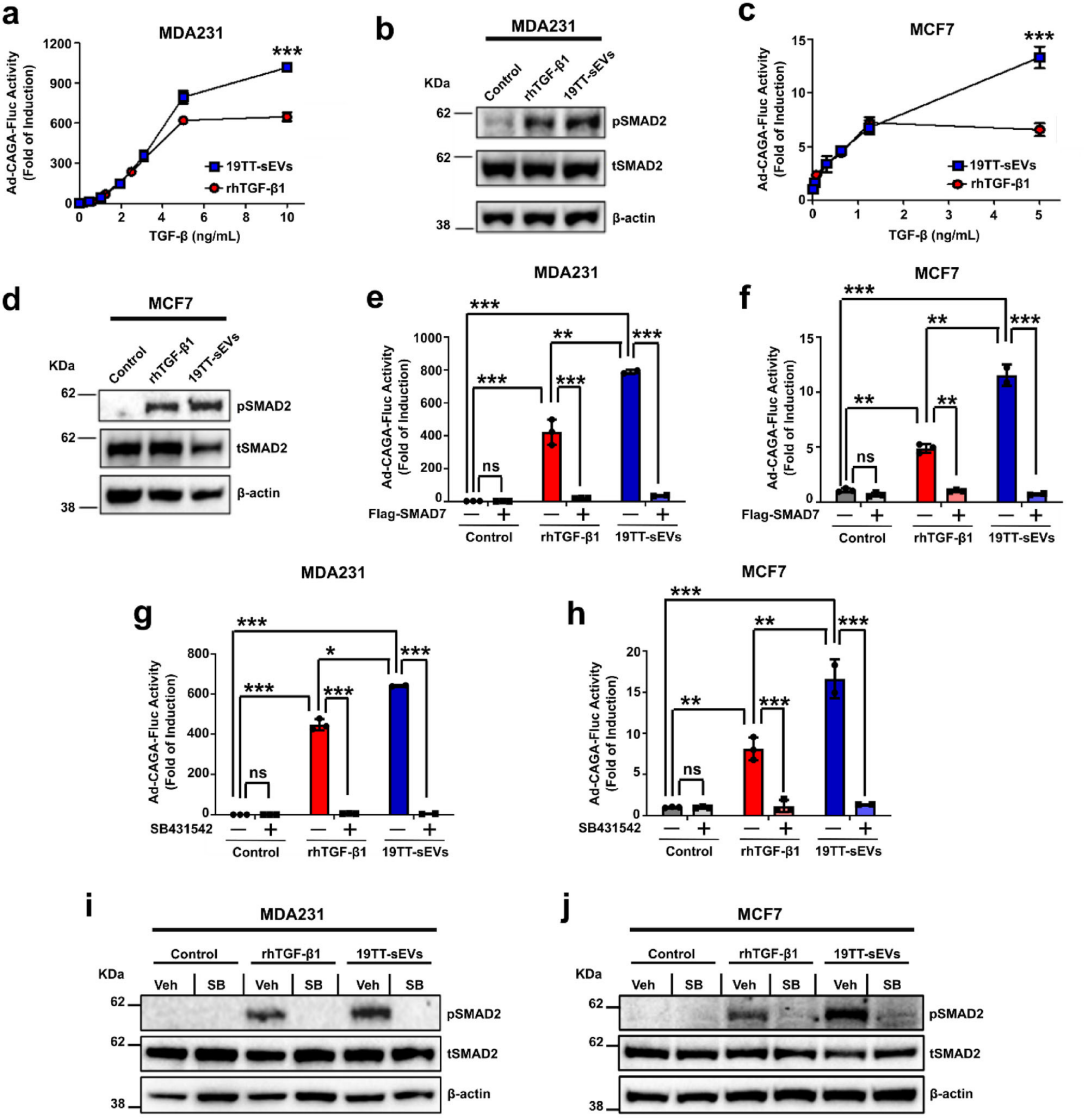
**CAF‐sEVs hyperactivate TGF‐β** signalling **in breast cancer cells in vitro. (a)** TGF/SMAD3 signalling reporter (Ad‐CAGA‐Fluc) activity was quantified in MDA231 cells treated with recombinant human (rh)TGF‐β1 or 19TT‐sEVs at increasing concentrations for 24 h. **(b)** Phosphorylated (p)SMAD2 levels analysed in MDA231 cells treated for 1 h. **(c)** TGF/SMAD3 signalling reporter (Ad‐CAGA‐Fluc) activity was quantified in MCF7 cells treated with rhTGF‐β1 or 19TT‐sEVs at increasing concentrations for 24 h. **(d)** pSMAD2 levels analysed in MCF7 cells treated for 1 h. **(e–f)** TGF/SMAD3 signalling reporter (Ad‐CAGA‐Fluc) activity was quantified in **(e)** MDA231 and **(f)** MCF7 cells infected with Ad‐CMV‐GFP (control adenovirus) or Ad‐CMV‐Flag‐SMAD7 treated for 24 h. **(g, h)** TGF/SMAD3 signalling reporter (Ad‐CAGA‐Fluc) activity was quantified in **(g)** MDA231 or **(h)** MCF7 cells challenged with SB431542 and treated with rhTGF‐β1 or 19TT‐sEVs for 24 h. **(i, j)** pSMAD2 levels in **(i)** MDA231 and **(j)** MCF7 challenged with SB431542 and treated with rhTGF‐β1 or 19TT‐sEVs for 1 h. DMSO was used as a vehicle for SB431542. The concentration of 19TT‐sEVs used to treat breast cancer cells in (b) and (d–j) was equivalent to 5 ng/mL TGF‐β activity. Results represent mean ± SD (*n* ≥ 3). Unpaired Student's *t*‐test was used to analyse data in **(a & c)**. One‐way ANOVA test followed by Dunn's Multiple Comparison test were used to analyse data in **(e–h)**. ns: statistically non‐significant, *p < 0.05, **p < 0.01, ***p < 0.001.

Next, we compared CAF‐sEVs’ ability to induce TGF‐β signalling activation with rhTGF‐β1. To this aim, highly metastatic MDA‐MB‐231 (henceforward MDA231) and poorly metastatic MCF7 breast cancer cells were treated with increasing concentrations of rhTGF‐β1 or CAF‐sEVs and the TGF‐β/SMAD signalling activity was evaluated. As quantified by luciferase assay, TGF‐β/SMAD signalling activity increased in MDA231 cells treated with rhTGF‐β1 or CAF‐sEVs in a concentration‐dependent manner (Figure 2a). Interestingly, results obtained upon treatment with rhTGF‐β1 (maximum induction: ∼650‐fold) were exceeded by treatment with CAF‐sEVs (maximum induction: ∼1015‐fold) (Figure 2a). Similar results were also observed by western blot regarding phosphorylated (p)SMAD2 levels (Figure 2b). Moreover, whereas MCF7 cells typically show poor response to rhTGF‐β1 (maximum induction: ∼7‐fold), cells treated with CAF‐sEVs at matching concentrations of TGF‐β showed significantly higher SMAD3/4 transcriptional activity (maximum induction: ∼13‐fold) (Figure 2c). Reinforcing this result, CAF‐sEVs also increased SMAD2 phosphorylation in MCF7 cells (Figure 2d).

To further validate observed results, TGF‐β/SMAD signalling activity was quantified in SMAD7 overexpressing cancer cells as SMAD7 is a specific TGF‐β signalling inhibitor. As expected, cells infected with control adenovirus (Ad‐CMV‐GFP) and treated with rhTGF‐β1 or CAF‐sEVs showed increased SMAD3/4 transcriptional activity, which was dramatically inhibited by SMAD7 overexpression (Figure 2e,f). Similarly, treatment with SB431542 (small molecule TβRI kinase inhibitor) abolished the effects triggered by rhTGF‐β1 or CAF‐sEVs (Figure 2g,h). Consistently, CAF‐sEV‐induced pSMAD2 was also impaired in cancer cells challenged with SB431542 (Figure 2i,j). These results reinforce our previous study where sEVs hyperactivate the TGF‐β signalling in highly metastatic breast cancer cells in an autocrine manner (Teixeira et al. 2023). Furthermore, they expand the previously reported ability of CAF‐sEVs to induce TGF‐β signalling activation by establishing their potential to hyperactivate this important pro‐metastatic molecular pathway in breast cancer cells in a paracrine way.

### 3.3 Heparin and PNP‐Xyl Treatment Inhibit TGF‐β Signalling Activity Induced by CAF‐sEVs in Breast Cancer Cells In Vitro

**FIGURE 3:**
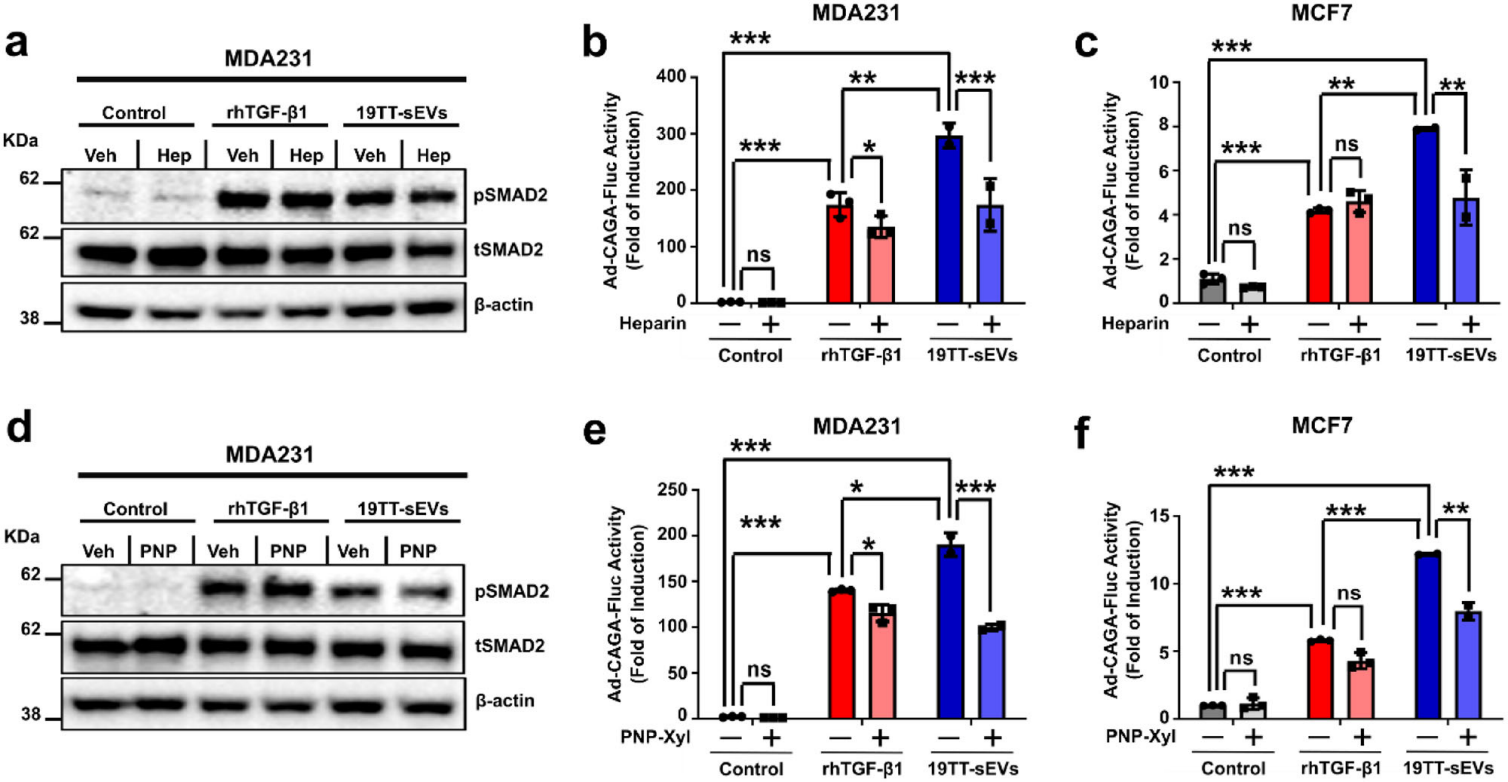
**Heparin and PNP‐Xyl treatment inhibit TGF‐β** signalling **activity induced by CAF‐sEVs in breast cancer cells in vitro. (a–c)** Effects caused by Heparin on cells treated with 1 ng/mL recombinant human (rh)TGF‐β1 or 19TT‐sEVs. **(a)** Phosphorylated (p)SMAD2 levels in MDA231 cells treated for 1 h. TGF‐β/SMAD signalling reporter (Ad‐CAGA‐Fluc) activity in **(b)** MDA231 or **(c)** MCF7 cells treated for 24 h. **(d‐f)** Effects caused by PNP‐Xyl on cells treated with 1 ng/mL rhTGF‐β1 or 19TT‐sEVs. **(d)** pSMAD2 levels in MDA231 cells treated for 1 h. TGF‐β/SMAD signalling reporter (Ad‐CAGA‐Fluc) activity in **(e)** MDA231 or **(f)** MCF7 cells treated for 24 h. The concentration of 19TT‐sEVs used to treat breast cancer cells was equivalent to 1 ng/mL TGF‐β activity. Results represent mean ± SD (*n* = 3). One‐way ANOVA test followed by Dunn's Multiple Comparison test were used to analyse data. ns: statistically non‐significant, *p < 0.05, **p < 0.01, ***p < 0.001.

Heparin and 4‐nitrophenyl β‐D‐xylopyranoside (PNP‐Xyl) have been described to impair EV uptake (Christianson et al. 2013; Franzen et al. 2014). To further evaluate EV‐induced effects, we treated breast cancer cells with these drugs prior treatment with CAF‐sEVs. MDA231 cells challenged with Heparin showed reduced pSMAD2 levels 1 h after treatment with CAF‐sEVs when compared with vehicle‐treated cells, an alteration not seen in counterparts treated with rhTGF‐β1 (Figure 3a). MDA231 cells also showed reduced SMAD3/4 transcriptional activity upon treatment with Heparin in cell cultures treated with rhTGF‐β1 or CAF‐sEVs for 24 h (Figure 3b), although this difference was more significant in cell cultures treated with the latter. In MCF7 cells, Heparin impaired the TGF‐β signalling in cell cultures treated with CAF‐sEVs, but such impact was not observed in cells treated with rhTGF‐β1 (Figure 3c). Validating this observation, similar results were observed in breast cancer cells treated with PNP‐Xyl (Figure 3d–f).

These results support the specificity of the effects induced by CAF‐sEVs and highlight the potential of targeting EV trafficking for the reduction of TGF‐β signalling levels in cancer cells. It is also noteworthy that while Heparin and PNP‐Xyl decreased CAF‐sEV‐induced TGF‐β signalling activity in both MDA231 and MCF7 cells, only MDA231 cells were affected by drug treatment in rhTGF‐β1‐treated cell cultures. These results imply an important difference in the biology of breast cancer cells regarding the mechanisms that regulate TGF‐β signalling levels. Whereas CAF‐sEVs hyperactivate the TGF‐β signalling in both highly and poorly metastatic cells, the former may engage its own EV trafficking machinery to sustain high TGF‐β signalling activity, while the latter cannot achieve similar outcomes due to lower EV secretion levels, as previously reported by our group (Teixeira et al. 2023).

### 3.4 CAF‐sEVs Rely on TGF‐β Signalling Activation to Induce Breast Cancer Cell Aggressiveness In Vitro

**FIGURE 4:**
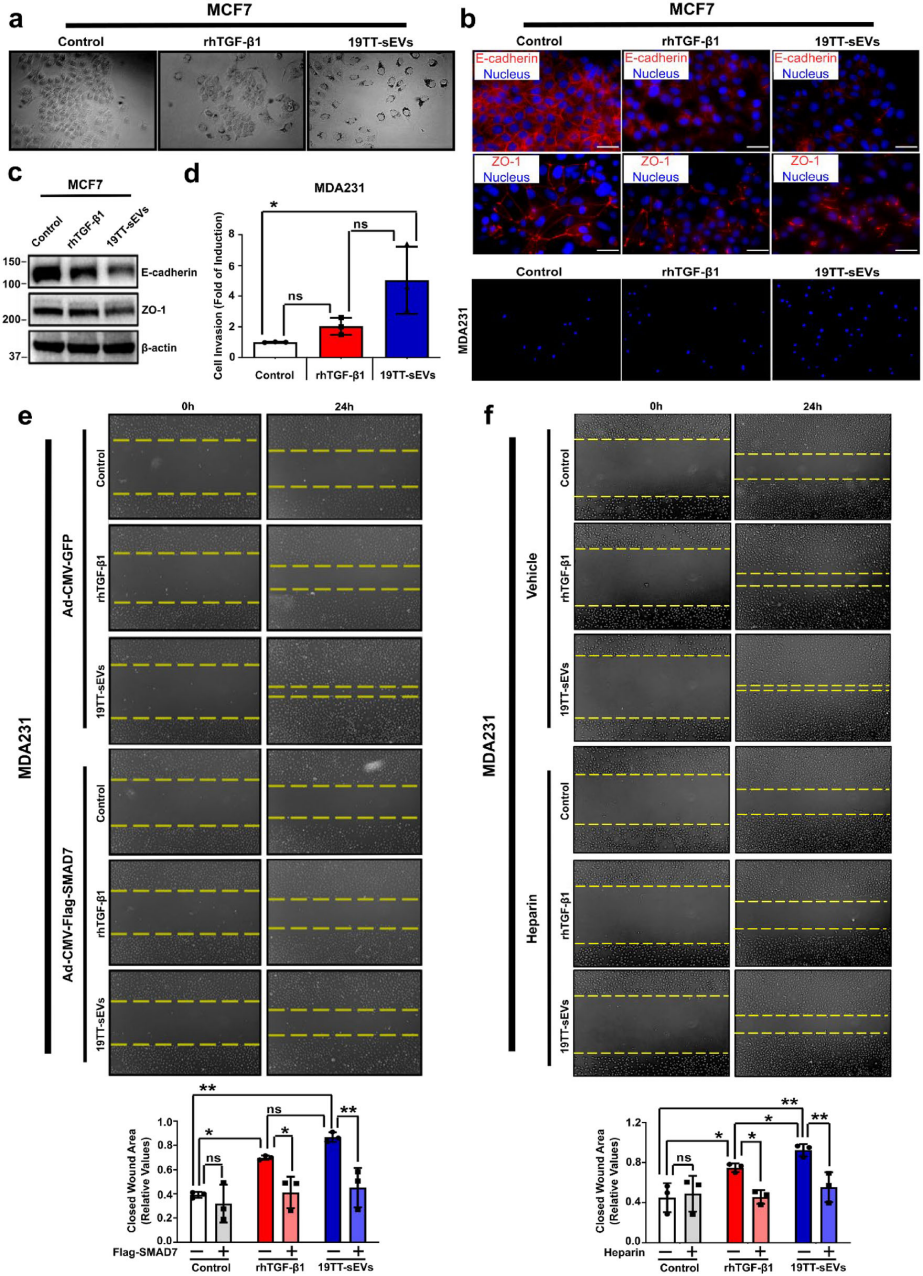
**CAF‐sEVs rely on TGF‐β** signalling **activation to induce breast cancer cell aggressiveness in vitro. (a)** MCF7 cell morphology analysed by bright field microscopy in cells treated with recombinant human (rh)TGF‐β1 or 19TT‐sEvs over 5 days. (20× magnification). Cell cultures were treated thrice (0, 48, and 96 h). Images obtained in cell cultures fixed and permeabilized. **(b)** E‐cadherin and ZO‐1 localization in MCF7 cells treated as in **(a)**. **(c)** E‐cadherin and ZO‐1 expression in MCF7 cells treated as in **(a)**. **(d)** MDA231 cell invasion in transwell inserts. **(e–f)** Wound healing assay for MDA231 cells challenged with **(e)** SMAD7 overexpression or **(f)** Heparin treatment. Ad‐CMV‐GFP: control adenovirus. The concentration of 19TT‐sEVs used to treat breast cancer cells was equivalent to 5 ng/mL TGF‐β activity. Results represent mean ± SD (*n* = 3). One‐way ANOVA test followed by Dunn's Multiple Comparison test were used to analyse data. ns: statistically non‐significant, *p < 0.05, **p < 0.01, ***p < 0.001.

Next, we examined whether CAF‐sEVs could induce a TGF‐β‐related pro‐metastatic phenotype in these cells. Indeed, while untreated MCF7 cells formed large clusters of cobblestone‐like cells, cells treated with rhTGF‐β1 or CAF‐sEVs showed scattered distribution and elongated phenotype (Figure 4a). Likewise, rhTGF‐β1‐treated MCF7 cells showed relocalization and downregulation of the epithelial markers E‐cadherin and zonula occludens 1 (ZO‐1). These alterations were further potentiated upon CAF‐sEV treatment (Figure 4b,c), demonstrating that CAF‐sEVs potently promote epithelial‐mesenchymal transition (EMT).

Furthermore, while highly metastatic MDA231 cells treated with rhTGF‐β1 showed elevated migration compared with untreated controls, this effect was enhanced in cell cultures treated with CAF‐sEVs (Figure S2a,b). Accordingly, treatment with CAF‐sEVs has also significantly increased MDA231 cell invasion (Figure 4d). Strikingly, while MCF7 cells show weak response to rhTGF‐β1, treatment with CAF‐sEVs enabled the migration and invasion of these poorly metastatic cells (Figure S2c–e). Confirming these observations, SMAD7 overexpression or SB431542 treatment nearly abolished breast cancer cell migration otherwise induced by CAF‐sEVs (Figures 4e and S3a–c).

Additionally, heparin treatment decreased the migration of MDA231 cells treated with rhTGF‐β1 or CAF‐sEVs (Figure 4f), while reducing the migration of MCF7 cells treated with CAF‐sEVs but not impacting rhTGF‐β1‐treated MCF7 cells (Figure S4a). Similar patterns were also observed in cell cultures challenged with PNP‐Xyl (Figure S4b,c).

Overall, our data confirm that sEVs secreted by CAFs are critical to breast cancer cell metastatic phenotype. In addition to hyperactivate the TGF‐β signalling, CAF‐sEVs potentiate the aggressiveness of highly invasive breast cancer cells and enable a similar behaviour in otherwise poorly invasive breast cancer cells.

### 3.5 CAFs Require Intact sEV Secretion to Hyperactivate the TGF‐β Signalling in Poorly Metastatic Breast Cancer Cells In Vitro

**FIGURE 5:**
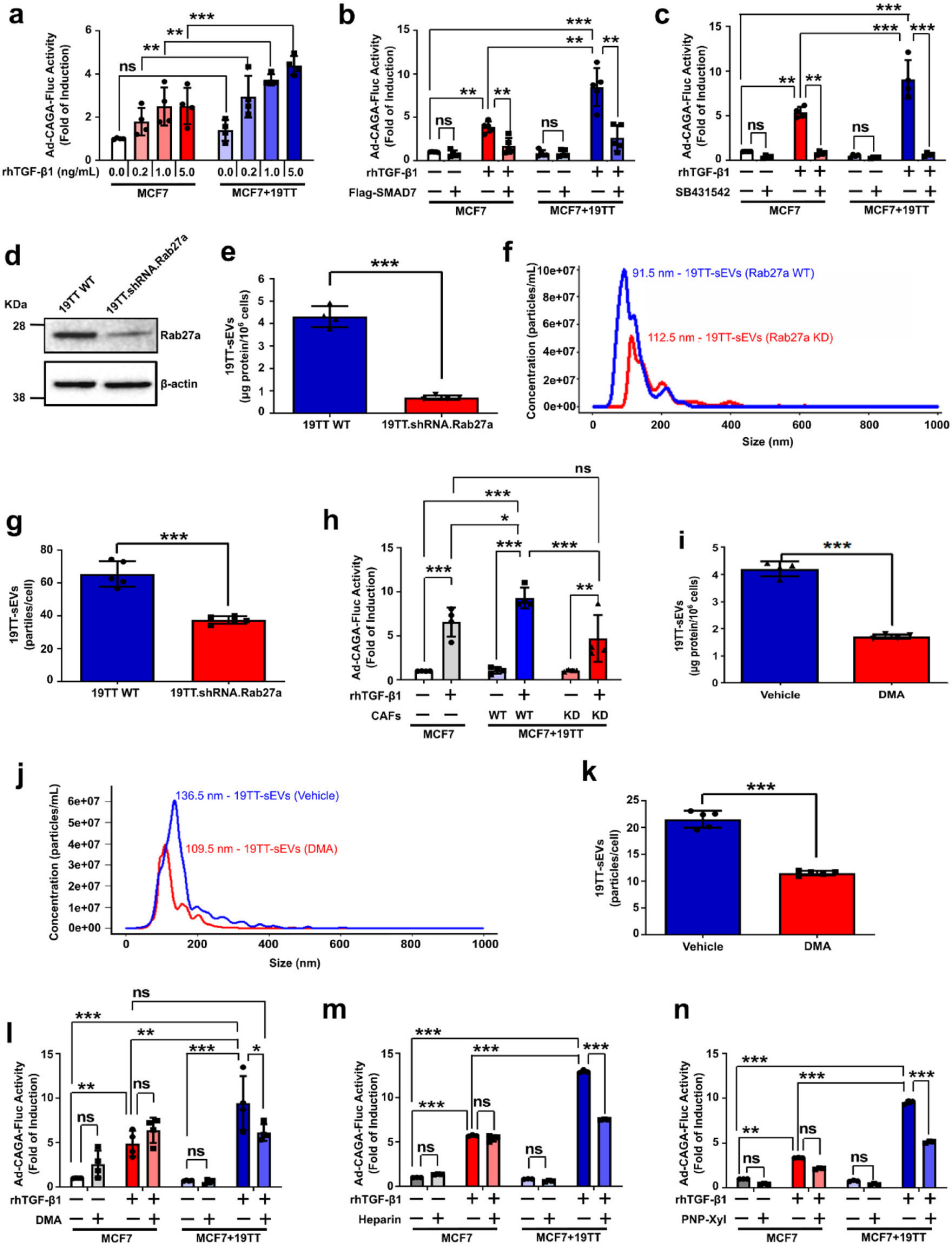
**CAFs require intact sEV secretion to hyperactivate the TGF‐β** signalling **in poorly metastatic breast cancer cells in vitro. (a–c)** TGF‐β/SMAD signalling reporter (Ad‐CAGA‐Fluc) activity in MCF7 cells ± 19TT cells treated as indicated. MCF7 cells were challenged with **(b)** SMAD7 overexpression or **(c)** SB431542 treatment. Ad‐CMV‐GFP: control adenovirus. DMSO: vehicle for SB431542. **(d)** Rab27a expression in parental (WT) and Rab27a knockdown (KD) 19TT cells. **(e)** Total sEV secretion quantified by BCA assay in 19TT‐sEVs. **(f)** Particle size distribution evaluated by nanoparticle tracking analysis (NTA) in 19TT‐sEVs. **(g)** Quantification of 19TT‐sEV secretion by NTA. **(h)** TGF‐β/SMAD signalling reporter (Ad‐CAGA‐Fluc) activity quantified as in **(A)** in MCF7 cells co‐cultured with 19TT (Rab27a WT or Rab27a KD) cells. **(i)** Total sEV secretion quantified by BCA assay in 19TT‐sEVs treated with DMA for 2 h. **(j)** Particle size distribution evaluated by NTA in 19TT‐sEVs treated as in **(i)**. **(k)** Quantification of sEV secretion by NTA in 19TT cells treated as in **(i)**. **(l‐n)** TGF‐β/SMAD signalling reporter (Ad‐CAGA‐Fluc) activity analysed as in **(A)** in MCF7 cells in cell cultures treated with **(l)** DMA, **(m)** Heparin, or **(n)** PNP‐Xyl for 24 h. Results represent mean ± SD (*n* ≥ 3). DMSO: vehicle for DMA and PNP‐Xyl. One‐way ANOVA test followed by Dunn's Multiple Comparison test were used to analyse data in **(a–c, h & l–n)**. Unpaired Student's *t*‐test was used to analyse data in **(e, g, i & k)**. ns: statistically non‐significant, *p < 0.05, **p < 0.01, ***p < 0.001.

After establishing that CAF‐sEVs induce TGF‐β signalling hyperactivation in breast cancer cells to enhance their metastatic potential in vitro, we sought to investigate if such effects could also be promoted in co‐cultures with CAFs and breast cancer cells (Figure S5a). Interestingly, we could not detect significant impact on MDA231 cells co‐cultured with CAFs (Figure S5b). As mono‐cultures of MDA231 cells treated with rhTGF‐β1 show very high TGF‐β/SMAD signalling activity, it might be possible that CAFs are simply not able to potentiate this effect in co‐cultures, regardless of rhTGF‐β1 treatment. Consequently, challenging with TGF‐β signalling inhibitors reduced the TGF‐β/SMAD signalling activity in MDA231 cells irrespective of co‐culturing with 19TT CAFs (Figure S5c,d). Contrarily, when compared with results obtained in mono‐cultures, TGF‐β/SMAD signalling levels were elevated in MCF7 cells co‐cultured with CAFs (Figure 5a). Noteworthy, while the TGF‐β signalling activity reached an expected plateau in mono‐cultures treated with ≥1 ng/mL rhTGF‐β1, this was not seen in MCF7 cells co‐cultured with CAFs (Figure 5a). In fact, the SMAD3/4 transcriptional activity in MCF7 cells co‐cultured with CAFs and treated with rhTGF‐β1 increased in a concentration‐dependent manner (Figure 5a), as seen for MCF7 cells treated with isolated CAF‐sEVs (Figure 2c). Further, TGF‐β signalling inhibitors efficiently decreased CAF‐promoted effects on MCF7 cells (Figure 5b,c).

To establish the contribution of EVs to CAF‐induced TGF‐β signalling hyperactivation, we generated stable Rab27 knockdown (KD) in 19TT CAFs (19TT.shRNA.Rab27a). Rab27a is critically involved in EV secretion by acting on the docking and fusion of multivesicular bodies to the plasma membrane (Bobrie et al. 2012; Ostrowski et al. 2010). After confirming reduced Rab27a levels and sEV secretion by Rab27a KD cells (Figure 5d–g), we compared the impact caused by CAFs (parental or Rab27a KD) on breast cancer cells. Like parental CAFs, Rab27a KD CAFs did not change the TGF‐β/SMAD signalling activity in MDA231 cells (Figure S5e). Nonetheless, the TGF‐β signalling in MCF7 cells co‐cultured with Rab27a KD CAFs was significantly decreased in comparison with cancer cells co‐cultured with parental CAFs (Figure 5h). As the TGF‐β signalling in MCF7 cells was comparably low in mono‐cultures and co‐cultures with Rab27a KD CAFs (Figure 5h), this result demonstrates that EV secretion is indeed crucial for the effects induced by CAFs on poorly metastatic breast cancer cells.

We have recently observed that treatment with the small molecule DMA inhibits the secretion of sEVs by MDA231 cells, impairing the autocrine activation of TGF‐β signalling in these breast cancer cells in vitro and in vivo (Teixeira et al. 2023). Aiming at expanding the therapeutic options to target EV‐induced TGF‐β signalling activity, we analysed if DMA could also reduce 19TT‐sEV secretion and impair the paracrine activation of the TGF‐β signalling in breast cancer cells by CAFs. DMA treatment decreased the secretion of sEVs by CAFs without major alterations in the particle size distribution of isolated sEVs (Figure 5i–k). Reinforcing our hypothesis that a reduced EV trafficking in MCF7 cells is not sufficient to affect TGF‐β signalling levels, DMA did not change the TGF‐β/SMAD signalling activity in MCF7 mono‐cultures (Figure 5l). Yet, while the SMAD3/4 transcriptional activity was increased in vehicle‐treated MCF7 cells co‐cultured with CAFs, this effect was impaired upon DMA challenging (Figure 5l). Moreover, treatment with Heparin or PNP‐Xyl also inhibited CAF‐induced TGF‐β signalling activity in MCF7 cells without significantly impacting mono‐cultures (Figure 5m‐5n). Confirming previous results, drugs targeting EV trafficking similarly inhibited the TGF‐β signalling in MDA231 cells regardless co‐culturing with CAFs (Figure S5f–h). These results demonstrate that EVs are critical mediators in the communication between CAFs and breast cancer cells and crucially involved in CAF‐induced TGF‐β signalling hyperactivation in MCF7 cells.

### 3.6 CAF‐Induced MCF7 Cell Aggressiveness Depends on EV Secretion and TGF‐β Signalling Pathway Activation

**FIGURE 6:**
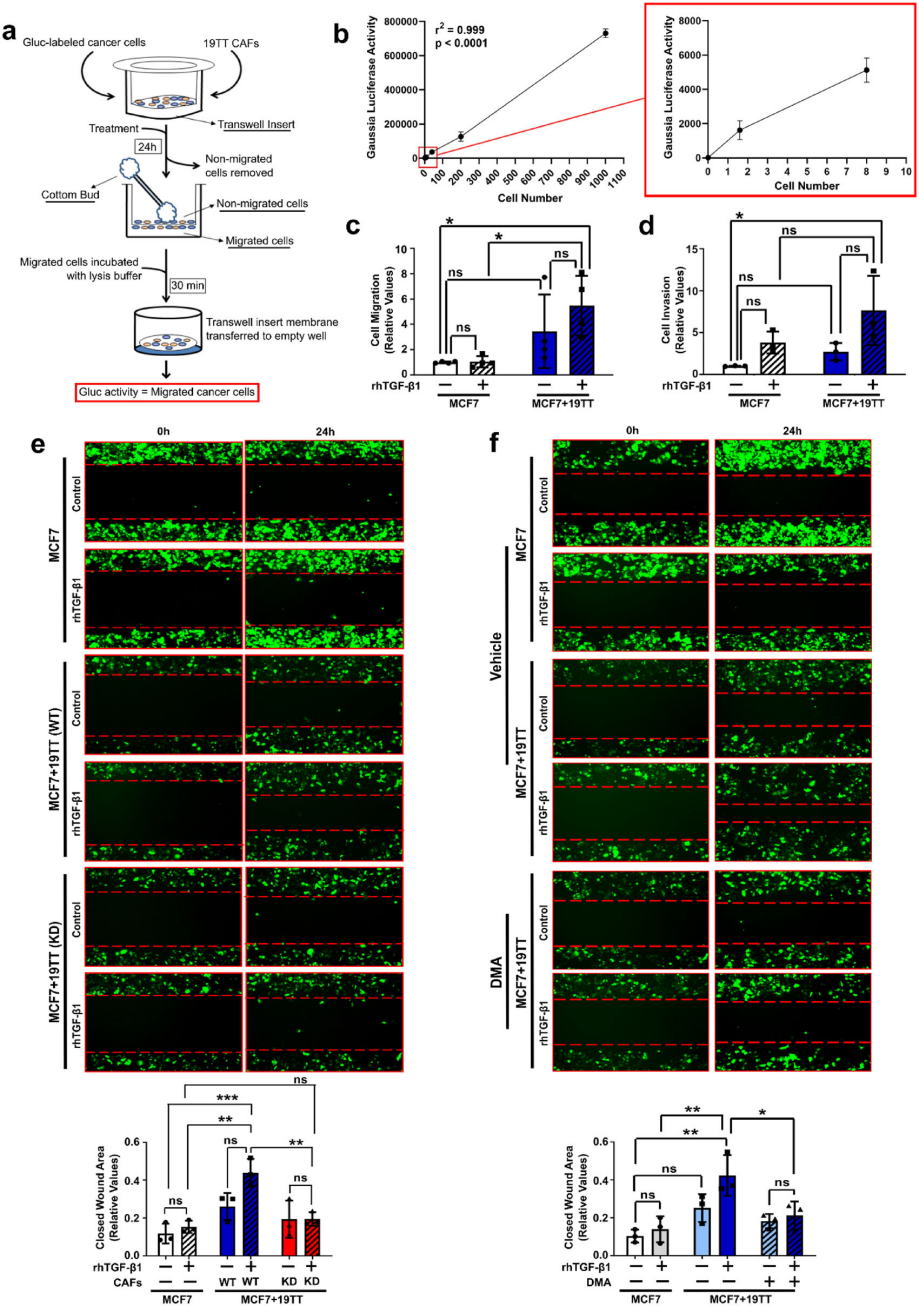
**CAF‐induced MCF7 cell aggressiveness depends on EV secretion and TGF‐β** signalling **pathway activation. (a)** Schematic illustration for the quantification of cancer cell migration in transwell inserts using Gaussia luciferase‐labelled MCF7 cells co‐cultured ± 19TT CAFs. Alternatively, transwell insert membranes were coated with Matrigel to evaluate cancer cell invasion. **(b)** Titration of Gaussia luciferase activity by luciferase assay using increasing numbers of Gaussia luciferase‐labelled MCF7 (MCF7.Gluc) cells. **(c)** Migration and **(d)** invasion of Gaussia luciferase‐labelled MCF7 (MCF7.Gluc) cells cultured ± 19TT cells and treated ± recombinant human (rh)TGF‐β1. **(e)** Wound healing assay for GFP‐labelled MCF7 cells cultured with 19TT (Rab27a WT or Rab27a KD) and treated as indicated. **(f)** Wound healing assay for GFP‐labelled MCF7 cells treated with vehicle (DMSO) or DMA. Results represent mean ± SD (*n* ≥ 3). One‐way ANOVA test followed by Dunn's Multiple Comparison test were used to analyse the presented data. ns: statistically non‐significant, *p < 0.05, **p < 0.01, ***p < 0.001.

Next, to investigate whether CAF‐increased TGF‐β signalling could enhance breast cancer cell aggressiveness, we established a model where Gaussia luciferase‐labelled MCF7 (MCF7.Gluc) breast cancer cells were co‐cultured with 19TT CAFs on top of transwell insert‐membranes (Figure 6a). Importantly, considering the high activity shown by Gaussia luciferase and the stable and constitutive expression of this enzyme in labelled cells, this model enables the detection and analysis of migratory (or invasive) breast cancer cells at single cell level (Figure 6b). In agreement with CAF‐induced hyperactivation of the TGF‐β signalling in MCF7 cells, increased migration and invasion were observed for MCF7 cells co‐cultured with CAFs and treated with rhTGF‐β1 (Figure 6c,d). This pattern was validated by wound healing assays using GFP‐labelled cancer cells (Figures S6 and S7). Moreover, we confirmed the requirement of an active TGF‐β signalling in breast cancer cells by showing that CAF‐induced MCF7 migration is impaired in SMAD7 overexpressing cancer cells (Figure S8a) and by SB431542 treatment (Figure S8b).

To ascertain the contribution of EVs to CAF‐induced MCF7 migration, we co‐cultured GFP‐labelled MCF7 cells with wild type (parental) or Rab27a KD 19TT CAFs. Supporting the important role played by EVs, parental CAFs efficiently enhanced MCF7 cell migration, while these cancer cells were not affected by co‐culture with Rab27a KD CAFs (Figure 6e). Further, challenging co‐cultures with DMA, Heparin or PNP‐Xyl has also led to slower migration of GFP‐labelled MCF7 cells in comparison with vehicle‐treated counterparts (Figure 6f and S9).

### 3.7 CAF‐sEVs Enhance TGF‐β Signalling Activity in MDA231 Cells In Vivo and Increase CTCs, Metastasis, and Tumour Self‐Seeding

**FIGURE 7:**
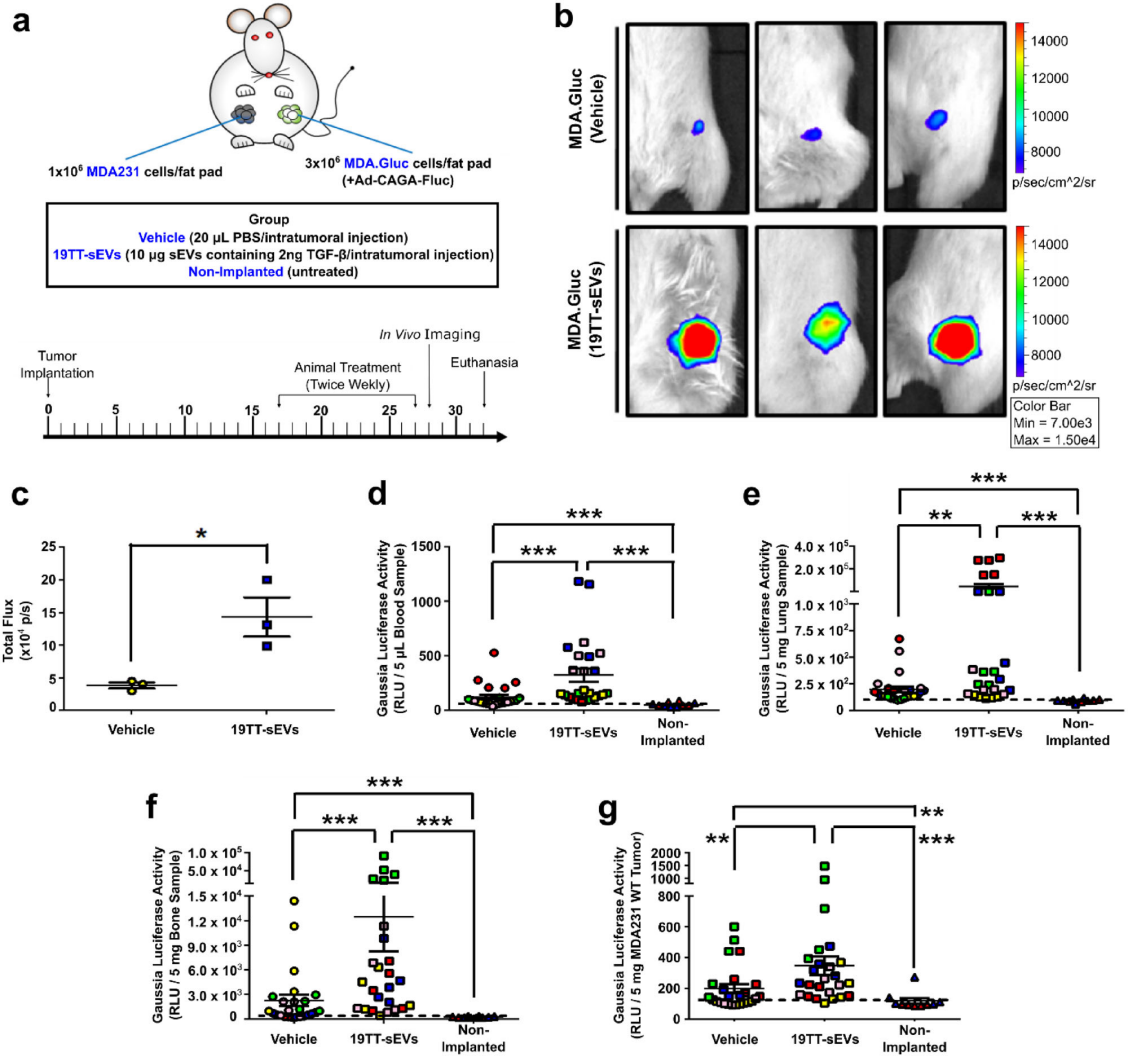
**CAF‐sEVs enhance TGF‐β** signalling **activity in MDA231 cells *in vivo* and increase CTCs, metastasis, and** tumour **self‐seeding. (a)** Schematic illustration and timeline for NOD‐SCID mice implanted with unlabelled MDA231 and Gaussia luciferase‐labelled MDA231 (MDA.Gluc) cells and treated ±19TT‐sEVs. **(b, c)** Quantification of TGF‐β/SMAD signalling reporter (Ad‐CAGA‐Fluc) activity in MDA.Gluc tumours by In Vivo Imaging System (*n* = 3 mice). **(d–g)** Gaussia luciferase activity in **(d)** blood, **(e)** lung, **(f)** bone, and **(g)** unlabelled MDA231 tumour samples (*n* = 5 mice). Animals are color‐coded. Black dashed lines indicate the background activity for the Gaussia luciferase quantified in samples from non‐implanted mice (*n* = 2 mice). Results represent mean ± SEM. Unpaired Student's *t*‐test **(c)**, One‐Way ANOVA followed by Tukey's Multiple Comparison Test **(d–g)**, *p < 0.05, **p<0.01, ***p<0.001, ns: statistically non‐significant.

After demonstrating that CAF‐sEVs increase the TGF‐β signalling activity in breast cancer cells in vitro, we sought to validate these findings in vivo. Gaussia luciferase‐labelled MDA231 (MDA.Gluc) cells were orthotopically implanted in NOD‐SCID mice. MDA.Gluc tumours infected with a TGF‐β/SMAD signalling reporter (Ad‐CAGA‐Fluc) were injected with vehicle or 10 µg CAF‐sEV total protein (2 ng TGF‐β activity) and the TGF‐β/SMAD signalling activity was quantified by In Vivo Imaging System (IVIS) (Figure 7a). Tumours injected with CAF‐sEVs showed similar growth and weight compared with vehicle‐treated counterparts (Figure S10a–f). Nonetheless, the TGF‐β/SMAD signalling activity quantified in tumours treated with CAF‐sEVs increased 3.7‐fold compared with vehicle‐treated tumours (Figure 7b,c), supporting the hypothesis of CAF‐sEV‐induced TGF‐β signalling hyperactivation.

Ex vivo, tissues/organs were harvested to assess the presence of MDA.Gluc cells in blood samples (circulating tumour cells; CTCs) and secondary organs (metastatic cells). Of note, this experiment was specifically designed to detect metastatic cells early after seeding and colonization of distant sites when only few cells are expected. The elevated activity showed by Gaussia luciferase allows the detection of cancer cells in these sites at single‐cell level and helps to overcome limitations imposed by traditional methods whether the differential growth of metastatic lesions may impact the analysis (Ware et al. 2024). Tissues/organs harvested from mice not implanted with MDA.Gluc cells (tumour naïve) were used as negative controls. Interestingly, compared with non‐implanted mice, Gaussia luciferase activity was detected in most blood samples harvested from mice implanted with MDA.Gluc cells (Figures 7d and S10g), confirming their elevated invasive potential. Moreover, in agreement with CAF‐sEV‐induced TGF/β signalling hyperactivation in MDA.Gluc tumours, CTCs were significantly increased in mice whose tumours were injected with CAF‐sEVs (Figures 7d and S10g). Accordingly, CAF‐sEVs also enhanced lung (Figures 7e and S10h) and bone (Figures 7f and S10i) colonization by metastatic MDA.Gluc cells.

Next, we focused on the impacts caused by treatment with CAF‐sEVs on tumour self‐seeding by analysing unlabelled MDA231 tumours (Figure 7a). Similarly to metastasis, tumour self‐seeding is a phenomenon also associated with cancer progression that is characterized by the return of CTCs to the primary tumour (Kim et al. 2009). Interestingly, tumour self‐seeding levels were naturally elevated and Gaussia luciferase activity was detected in several samples from vehicle‐treated mice, and this process was further potentiated in CAF‐sEV‐treated animals (Figures 7g and S10j).

These results confirm that CAF‐sEVs can also amplify the TGF‐β signalling in highly metastatic MDA.Gluc breast cancer cells in vivo. Consequently, treatment with CAF‐sEVs enhanced the aggressiveness of MDA.Gluc tumours, increasing cancer progression.

### 3.8 TGF‐β Signalling Hyperactivation In Vivo by CAF‐sEVs Enables Progression of Poorly Metastatic MCF7 Cancer Cells

**FIGURE 8:**
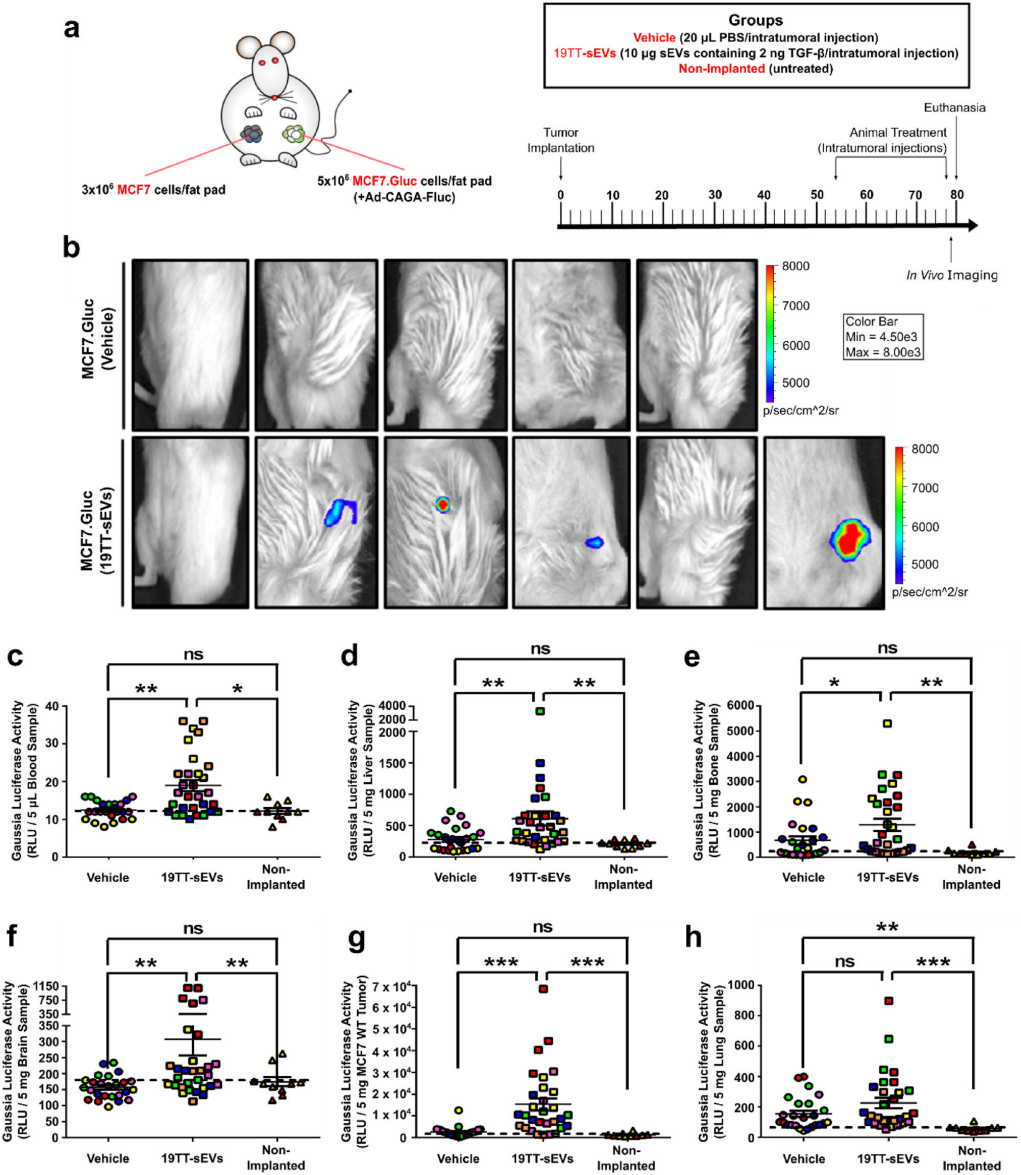
**TGF‐β** signalling **hyperactivation *in vivo* by CAF‐sEVs enables progression of poorly metastatic MCF7 cancer cells. (a)** Schematic illustration and timeline for NOD‐SCID mice implanted with unlabelled MCF7 and Gaussia luciferase‐labelled MCF7 (MCF7.Gluc) cells and treated ± 19TT‐sEVs. **(b)** Detection of TGF‐β/SMAD signalling reporter (Ad‐CAGA‐Fluc) activity in MCF7.Gluc tumours by In Vivo Imaging System (*n* = 5–6 mice). **(c–h)** Gaussia luciferase activity in **(c)** blood, **(d)** liver, **(e)** bone, **(f)** brain, **(g)** unlabelled MCF7 tumours and **(h)** lung samples (*n* = 5–6 mice). Animals are color‐coded. Black dashed lines indicate the background activity for the Gaussia luciferase quantified in samples from non‐implanted mice (*n* = 2 mice). Results represent mean ± SEM. One‐Way ANOVA followed by Tukey's Multiple Comparison Test. *p < 0.05, **p < 0.01, ***p < 0.001, ns: statistically non‐significant.

Although CAF‐sEVs efficiently amplified the TGF‐β signalling activity in MCF7 cells in vitro, we asked whether the limited responsiveness of these breast cancer cells to TGF‐β would restrict similar outcomes in vivo. To address this question, MCF7.Gluc cells were orthotopically implanted in NOD‐SCID mice, treated with vehicle or CAF‐sEVs, and the TGF‐β/SMAD reporter (Ad‐CAGA‐Fluc) activity was quantified by IVIS (Figure 8a). MCF7.Gluc tumours were not affected by treatment regarding growth or weight (Figure S11a–f). Surprisingly, while the TGF‐β/SMAD signalling was analysed in eleven tumours (one tumour/mouse), only four of these tumours showed bioluminescence above background levels, all of them among tumours injected with CAF‐sEVs (Figure 8b).

Next, we investigated if CAF‐sEVs could enable a metastatic phenotype in weakly invasive MCF7 breast cancer cells. Interestingly, while CTCs were only detected in a minority of blood samples harvested from vehicle‐treated mice, treatment with CAF‐sEVs significantly promoted MCF7.Gluc cell intravasation (Figures 8c and S11g). Consistently, whereas metastatic cells were only detected in few samples from vehicle‐treated animals, most samples from mice injected with CAF‐sEVs exhibited metastasis to liver (Figures 8d and S11h), bones (Figures 8e and S11i) and brain (Figures 8f and S11j) and tumour self‐seeding (Figures 8g and S11k). Surprisingly, most lung samples harvested from animals implanted with MCF7.Gluc cells showed detectable metastatic cells (Figures 8h and S11l), which could be partially attributed to the prolonged timeline of this experiment. These results demonstrate that CAF‐sEVs induce TGF‐β signalling hyperactivation in MCF7 cells in vivo to enable multiorgan metastasis and tumour self‐seeding.

### 3.9 CAF‐Induced TGF‐β Signalling Hyperactivation and Breast Cancer Progression Is Impaired by Genetically and Pharmacologically Targeting EV Trafficking

**FIGURE 9:**
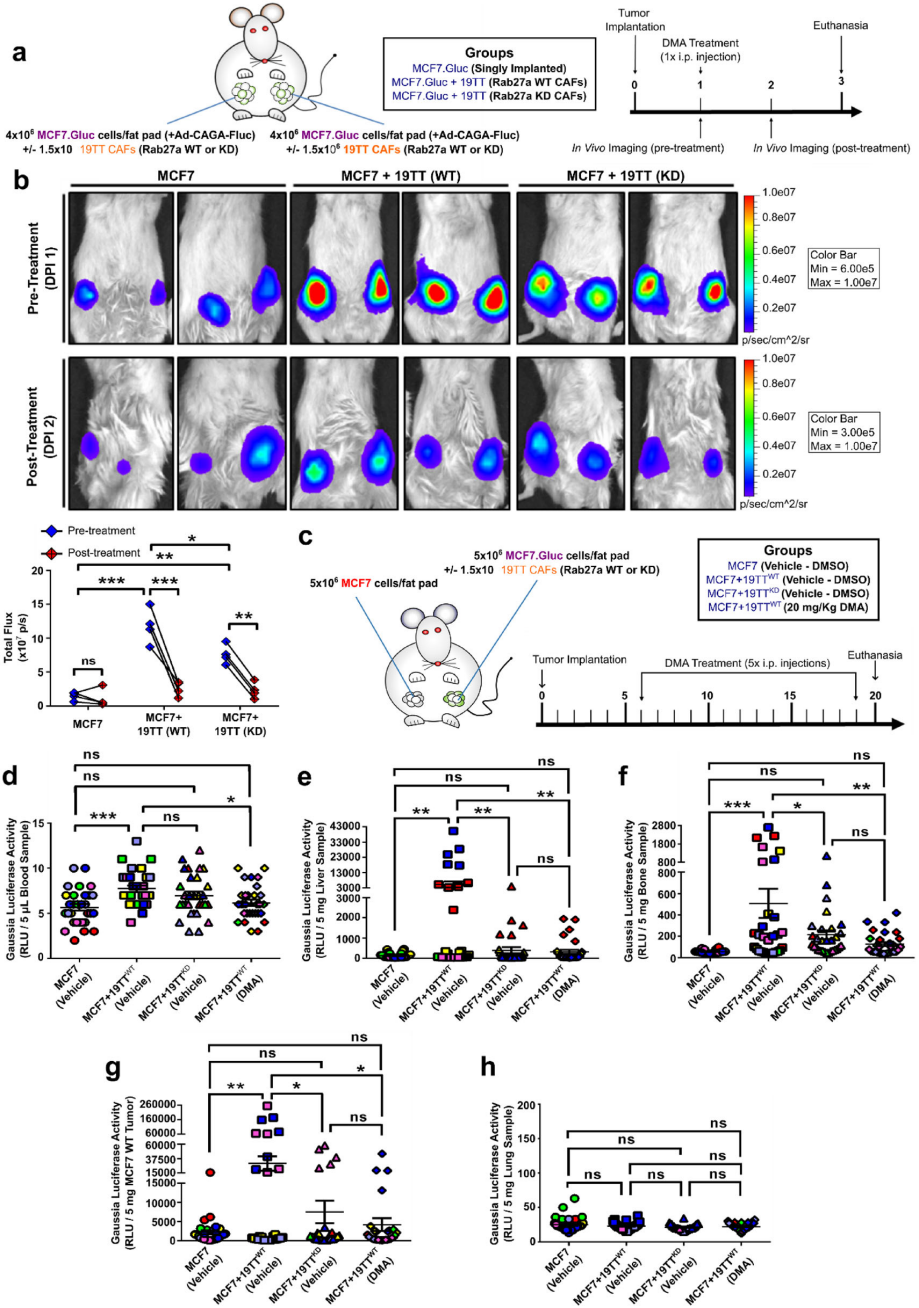
**CAF‐induced TGF‐β** signalling **hyperactivation and breast cancer progression is impaired by genetically and pharmacologically targeting EV trafficking. (a)** Schematic illustration and timeline for NOD‐SCID mice implanted with Gaussia luciferase‐labelled MCF7 (MCF7.Gluc) cells ±19TT (Rab27a wild type/WT or knockdown/KD). **(b)** Quantification of TGF‐β/SMAD3 signalling reporter (Ad‐CAGA‐Fluc) activity in MCF7.Gluc cells by In Vivo Imaging System (*n* = 2 tumour/mice; two mice/group). **(c)** Schematic illustration and timeline for NOD‐SCID mice implanted with unlabelled MCF7 cells and Gaussia luciferase‐labelled MCF7 (MCF7.Gluc) cells ±19TT (Rab27a WT or KD). **(d‐h)** Gaussia luciferase activity in **(d)** blood, **(e)** liver, **(f)** bone, **(g)** unlabelled MCF7 tumours, and **(h)** lung samples (*n* = 6 mice). Animals are color‐coded. Results represent mean ± SEM. One‐Way ANOVA followed by Tukey's Multiple Comparison Test. *p < 0.05, **p < 0.01, ***p < 0.001, ns: statistically non‐significant.

Our results have established sEVs as the main mechanisms used by CAFs to drive TGF‐β signalling hyperactivation in MCF7 cells in vitro. To elucidate the relevance of this mechanism in vivo, MCF7.Gluc cells were orthotopically implanted in NOD‐SCID mice either singly or in combination with CAFs. TGF‐β signalling activity was then quantified by IVIS according to the bioluminescence emitted by MCF7.Gluc cells infected with a TGF‐β/SMAD signalling reporter (Ad‐CAGA‐Fluc) prior to implantation (Figure 9a). Compared with singly implanted MCF7.Gluc cells, co‐implantation with Rab27a WT CAFs greatly increased the TGF‐β/SMAD signalling activity in MCF7.Gluc cells in vivo. Yet, a significant reduction in the bioluminescence emitted by MCF7.Gluc cells was quantified upon co‐implantation with Rab27a KD CAFs (Figure 9b). Immediately following the first set of in vivo imaging, mice were challenged with 20 mg/Kg DMA and re‐imaged 24 h after treatment (Figure 9a). DMA treatment efficiently impaired the TGF‐β/SMAD signalling activity in cancer cells co‐implanted with CAFs, reducing it to basal levels comparable to those detected in singly implanted MCF7.Gluc cells (Figure 9b), and confirming a major role for EVs in CAF‐induced effects.

Next, we analysed if the increased TGF‐β signalling activity levels seen in MCF7.Gluc cells in vivo that were attributed to CAF‐sEVs could be associated with altered metastatic potential. NOD‐SCID mice were orthotopically and contralaterally implanted with MCF7.Gluc cells ± 19TT cells and unlabelled MCF7 cells. Six days post‐implantation, animals were treated with vehicle or 20 mg/Kg DMA and cancer progression was assessed by *ex vivo* luciferase assay in samples harvested 20 days post‐implantation (Figure 9c). Tumour growth and weight were not affected by co‐implantation with CAFs or drug challenging within this short period (Figures S12a‐S12f). Yet, increased Gaussia luciferase activity was quantified in blood samples from mice co‐implanted with Rab27a WT CAFs, and this activity was reduced in mice injected with DMA (Figures 9d and S12g). However, considering the low bioluminescence detected among all samples, this difference may not accurately represent alterations in CTC numbers. Nevertheless, analysis of secondary organs determined augmented MCF7.Gluc metastasis in liver (Figures 9e and S12h) and bones (Figures 9f and S12i) and tumour self‐seeding (Figure 9g and S12j) in mice co‐implanted with Rab27a WT CAFs. Moreover, compared with animals singly implanted with MCF7.Gluc cells, no significant alterations were observed upon co‐implantation with Rab27a KD CAFs or DMA treatment regarding metastasis or tumour self‐seeding (Figures 9e–g and S12). Also, lung samples did not show changes in Gaussia luciferase activity irrespective of co‐implantation with CAFs or DMA treatment (Figure 9h and S12m).

**FIGURE 10:**
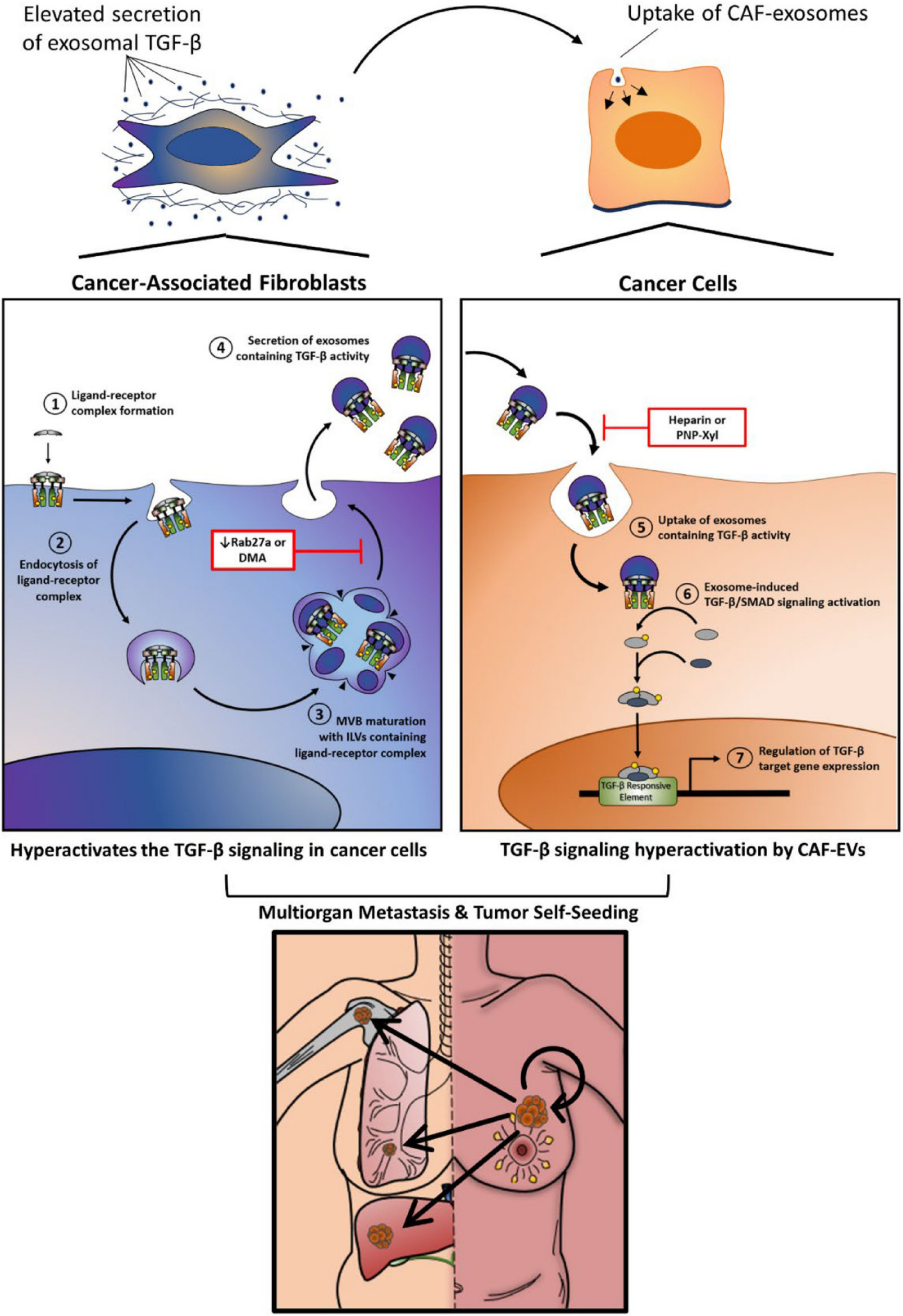
**Working model**. Human breast CAFs secrete elevated levels of vesicular TGF‐β. Uptake of CAF‐sEVs by breast cancer cells drives TGF‐β signalling hyperactivation to ultimately increase multiorgan metastasis and tumour self‐seeding. Consequently, decreasing Rab27a levels in CAFs reduces sEV secretion and prevents the amplification of TGF‐β signalling levels in breast cancer cells to ameliorate cancer progression. Accordingly, sEV trafficking disruption by treatment with DMA, heparin, or PNP‐Xyl is a novel therapeutic strategy that normalizes the TGF‐β signalling activity in breast cancer cells and efficiently blocks metastatic progression. In this illustration, arrow thickness represents the intensity of a given step. Red and white boxes highlight therapeutic strategies used in this work to normalize TGF‐β signalling levels by decreasing CAF‐sEV secretion and uptake. MVB: multivesicular body (also termed late endosome). ILVs, intraluminal vesicles.

Altogether, results presented here demonstrate that CAFs promote the progression of non‐invasive breast cancers by secreting sEVs to hyperactivate the TGF‐β signalling in breast cancer cells. Poorly invasive breast cancer cells exhibit low sEV secretion, which is insufficient to amplify TGF‐β signalling to the levels required for successful metastasis. Human breast CAFs, otherwise, secrete high levels of sEVs containing TGF‐β activity. Therefore, CAF‐sEVs can operate as a paracrine supply to boost the TGF‐β signalling in breast cancer cells. More specifically, CAF‐sEVs not only increase the TGF‐β signalling activity in non‐invasive breast cancer cells but lead to a hyperactivated state that enables cancer cell invasion, dissemination, and metastasis. Consequently, the precise characterization of the communication between CAFs and breast cancer cells allowed us to disrupt this process by blocking EV secretion/uptake within the TME. This strategy reduced the TGF‐β signalling activity in breast cancer cells to prevent their progression toward an aggressive phenotype, thus, impairing metastasis (Figure 10).

## 4 Discussion

The relevance of TGF‐β and its signalling pathway to cancer progression and poor patient outcome are well established (Ren et al. 2020; Javle et al. 2014; Ware and Zhu 2020), leading several TGF‐β signalling inhibitors to clinical trials (Liu and Ren 2021; Metropulos et al. 2022; Gulley et al. 2022). Yet, results obtained in cancer clinical studies have been disappointing as none of the pharmacological inhibitors tested so far succeeded in significantly prolonging the survival of cancer patients (Teixeira et al. 2020; Wu et al. 2023). This issue implies a major gap in our ability to reproduce the context and mechanisms regulating TGF‐β signalling levels in human cancers when evaluating the efficacy of TGF‐β signalling inhibitors in pre‐clinical cancer models. Here, we show that human breast CAFs are critical elements able to drive metastasis by secreting sEVs that hyperactivate the TGF‐β signalling in breast cancer cells. CAF‐sEVs potentiate the aggressiveness of highly metastatic breast cancer cells and induce metastasis in otherwise low aggressive breast cancer cells. Accordingly, genetically impairing the secretion of sEVs in CAFs reversed CAF‐induced effects in vitro and in vivo. Further advancing this finding, we devised a DMA‐based therapeutic approach that hindered the communication between CAFs and breast cancer cells to reduce TGF‐β signalling levels in breast cancer cells at the primary tumour and impair multiorgan metastasis.

Increasing evidence shows that non‐cancer cells within the TME critically impact cancer growth and metastasis (de Visser and Joyce 2023; Anderson and Simon 2020). CAFs stand out among other non‐cancer cells both in numbers and function since they represent the main cellular component of the tumour stroma and affect most cancer hallmarks (Sahai et al. 2020; Biffi and Tuveson 2021; Simon and Salhia 2022). In this context, our work determines a major significance for human breast CAFs by showing that their mere presence enables cancer progression, inducing a metastasis‐competent phenotype in poorly invasive breast cancer cells. Notably, rather than a simple consequence of the crosstalk between CAFs and breast cancer cells, CAF‐induced TGF‐β signalling hyperactivation is as a driving force required for breast cancer metastasis. Accordingly, both the specific inhibition of the TGF‐β signalling in breast cancer cells by SMAD7 overexpression and the treatment of cell cultures with the selective TβRI kinase inhibitor SB431542 abrogated CAF‐induced cancer cell aggressiveness. Whereas CAFs can modulate multiple pro‐metastatic molecular pathways (Yang et al. 2016; Jia et al. 2013; Zhang et al. 2017; Zhao et al. 2017; Ding et al. 2018; Yan et al. 2018; Hu et al. 2021; Liu et al. 2021; Ren et al. 2021; Sun and Chen 2021; Ma et al. 2022), our results point to TGF‐β as the root‐cause of breast cancer metastasis.

Moreover, although fibroblast‐derived EVs are described to transport TGF‐β (Li et al. 2017) and TβRII (Languino et al. 2016), their precise contribution to TGF‐β signalling activation remained unclear. In the present work, we demonstrate that most of the TGF‐β activity in the secretome of human breast CAFs resides in sEVs, which carry TGF‐β1, TβRI, TβRII, and SMAD2 at least. Consequently, our data show that sEVs are the main mediators used by CAFs to hyperactivate the TGF‐β signalling in breast cancer cells. Yet, the relevance of CAF‐sEVs differs between highly and poorly invasive breast cancer cells. In highly invasive breast cancer cells, termination of TGF‐β signalling by endocytosis and degradation of the TGF‐β receptor complex (Tzavlaki and Signaling 2020; Heldin and Moustakas 2016) is outweighed by an autocrine mechanism based on increased secretion and uptake of sEVs containing TGF‐β activity (Teixeira et al. 2023). Thus, breast cancer cells with elevated metastatic potential do not rely on CAF‐sEVs as their own sEVs suffice to hyperactivate the TGF‐β signalling (Teixeira et al. 2023). Contrarily, autocrine activation of the TGF‐β signalling in poorly invasive breast cancer cells is quickly followed by signalling termination (Wu et al. 2023) due to low sEV secretion (Teixeira et al. 2023). Nevertheless, we demonstrate that this could be compensated in a paracrine manner by CAF‐sEVs, which hyperactivate the TGF‐β signalling in poorly invasive breast cancer cells to enable metastasis. Although the precise mechanisms underlying TGF‐β signalling hyperactivation by CAF‐sEVs still require further dissection, this could be associated with the prolonged retention of sEVs into the endosomes of target cells, a phenomenon previously observed with mast cell‐sEVs (Shelke et al. 2019). Further, CAF‐sEVs might also target non‐cancer cells. Since TGF‐β is known to promote immunosuppression, myofibroblast activation, and angiogenesis (Teixeira et al. 2022), the uptake of CAF‐sEVs by non‐cancer cells is likely to further support metastasis by establishing a permissive microenvironment. Thus, the therapeutic targeting of EV trafficking may help to prevent cancer progression—and even potentiate the use of other anticancer therapies—by simultaneously reducing TGF‐β signalling levels across multiple tumour compartments.

Many drugs have been used to target EV trafficking (Christianson et al. 2013; Chalmin et al. 2010; Im et al. 2019; Atai et al. 2013; Chitti et al. 2022; Vader et al. 2014). Nevertheless, since most pre‐clinical cancer studies only report impacts on tumour growth, little is known about the efficiency of these drugs in blocking metastasis, particularly considering TME components. Our in vitro results show that treatment with DMA, Heparin, and PNP‐Xyl impair the impacts induced by CAFs on breast cancer cells, reducing TGF‐β signalling activity and cancer cell metastatic potential. These effects were recapitulated by *in vivo* treatment with DMA, where disrupting EV‐mediated communication between CAFs and cancer cells reduced TGF‐β signalling levels in the latter to potently block tumour self‐seeding and metastasis to liver and bones. Interestingly, we did not detect alterations in the growth of primary tumours in our co‐implantation models irrespective of DMA treatment. Defining whether this was due to the dose and duration of the treatment used in this work or to other biological variables will require additional investigation in future studies. Also, albeit DMA efficiently reduces CAF‐sEV secretion, effects associated with systemic administration must not be overlooked. We previously shown that DMA treatment reduces the metastatic dissemination of highly invasive breast cancer cells (Teixeira et al. 2023). Additionally, DMA might also affect other signalling pathways and cell types within the TME and in healthy tissues, reducing metastasis by additionally preventing the establishment of pre‐metastatic niches (Costa‐Silva et al. 2015; Garcia‐Silva et al. 2021; Hoshino et al. 2015; Ji et al. 2020; Morrissey et al. 2021; Murgai et al. 2017).

Altogether, our findings define a new role for CAF‐sEVs in promoting metastatic dissemination by driving TGF‐β signalling hyperactivation in breast cancer cells. Since increased TGF‐β signalling is required for breast cancer metastasis and CAF‐sEVs suffice to hyperactivate this molecular pathway in breast cancer cells, CAFs are established as a crucial component of the breast TME. Accordingly, our study helps to explain the failure in translating TGF‐β signalling inhibitors into clinical care when considering the historical overlooking of human CAFs in pre‐clinical cancer models. In this regard, we devised a novel and more effective therapeutic strategy to inhibit TGF‐β signalling in breast cancer cells. Instead of targeting TGF‐β signalling components, inhibition of the EV trafficking within the TME was conceived to disable the communication between CAFs and breast cancer cells, blocking CAF‐sEVs as source of TGF‐β, and preventing breast cancer metastasis. Thus, this work presents a compelling argument for further development and translation of inhibitors targeting EV secretion and uptake into cancer clinical trials as antimetastatic therapies.

## AUTHOR CONTRIBUTIONS

**Adilson Fonseca Teixeira**: formal analysis (lead), investigation (lead), methodology (supporting), writing–original draft (lead), writing–review and editing (lead). **Yanhong Wang**: methodology (Supporting). **Josephine Iaria**: investigation (supporting), methodology (supporting), resources (lead). **Peter ten Dijke**: supervision (supporting), writing–review and editing (supporting). **Hong‐Jian Zhu**: conceptualization(Lead), funding acquisition(Lead), methodology(Lead), supervision(Lead), writing–review and editing(Supporting).

## Ethics Statement

All in vivo experiments were done in agreement with the National Health Medical Research Council of Australia code of practice for the care and use of animals for scientific purposes. Use of animals for experimental purposes was approved by The University of Melbourne Animal Ethics Committee (Ethics ID: 1914917).

## Conflicts of Interest

The authors report no conflict of interest. J.I., A.F.T and H.‐J.Z. are members of the research team at the Huagene Institute. The Huagene Institute had no role in the design of the study; in the collection, analyses, or interpretation of data; in the writing of the manuscript, or in the decision to publish the results.

## Supporting information



Supporting Information

## Data Availability

All data supporting the findings of this study are available within the article and as supplemental material. Raw data and materials are available from the corresponding authors upon reasonable request.
