# Supplementary material for: Extracellular Vesicles Secreted by Cancer‐Associated Fibroblasts Drive Non‐Invasive Cancer Cell Progression to Metastasis via TGF‐β Signalling Hyperactivation
Source: J Extracell Vesicles. 2025 Mar 16;14(3):e70055. doi: 10.1002/jev2.70055 (PMC11911544; doi:10.1002/jev2.70055)
Supplement: Supplementary file 1 — Supporting Information [file JEV2-14-e70055-s001.pdf]

Supplementary Information

Extracellular vesicles secreted by cancer-associated fibroblasts drive non-invasive cancer cell progression to metastasis via TGF-β signaling hyperactivation

Adilson Fonseca Teixeira[1,2], Yanhong Wang[1], Josephine Iaria[1,2], Peter ten Dijke[3], and Hong-Jian Zhu[1,2, *]

[1]Department of Surgery (The Royal Melbourne Hospital), The University of Melbourne, Parkville, Victoria, Australia

[2]Huagene Institute, Kecheng Science and Technology Park, Pukou District, Nanjing 211806, Jiangsu, China

[3]Department of Cell and Chemical Biology, Oncode Institute, Leiden University Medical Center, Leiden, The Netherlands

[*]**Correspondence:** Department of Surgery, The University of Melbourne, 5th Floor Clinical Sciences Building, The Royal Melbourne Hospital, Parkville 3050, Australia (Tel: 61 3 8344 3025; Fax: 61 3 9347 6488, email: hongjian@unimelb.edu.au)

Supplementary Figures

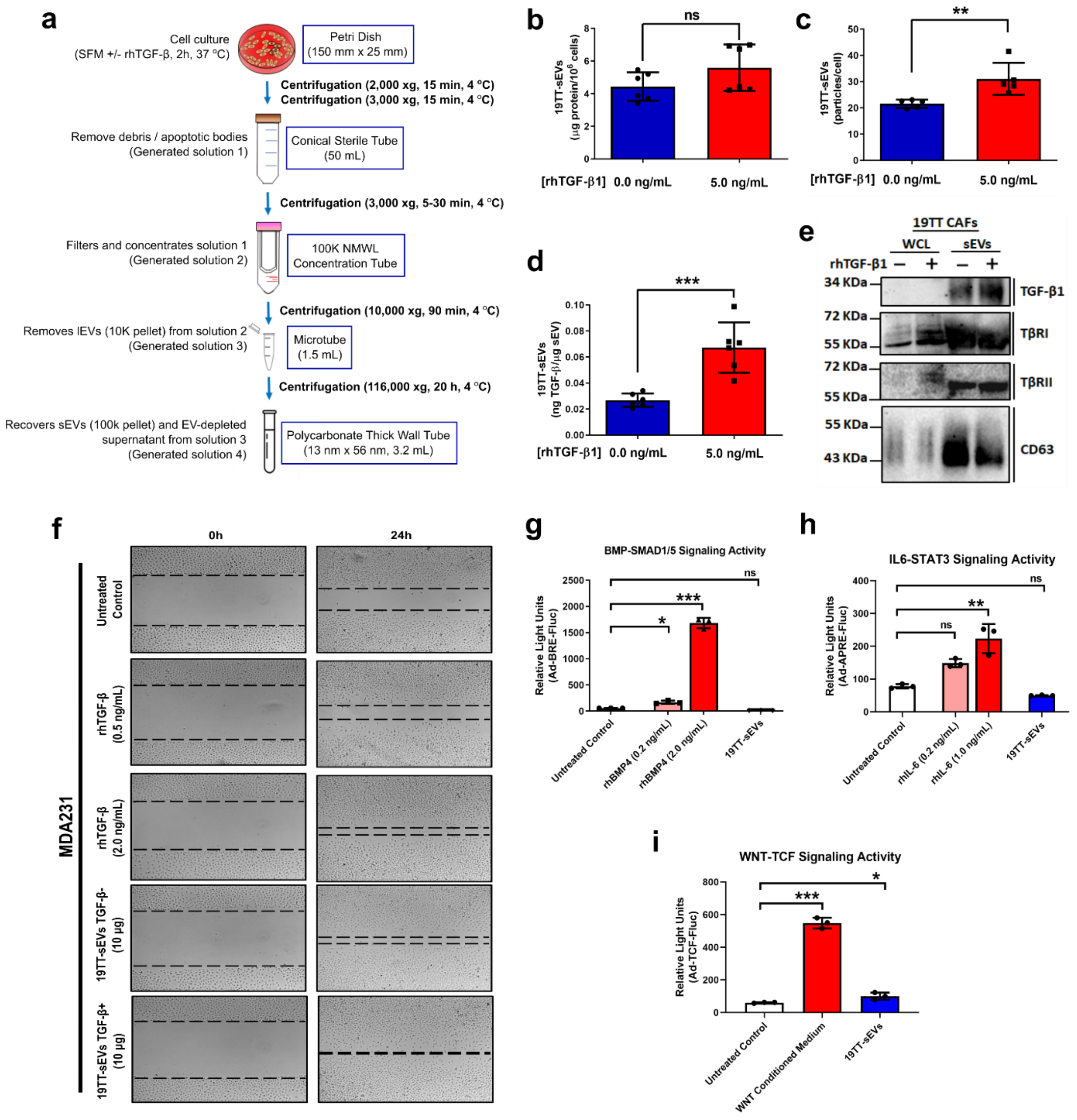

Figure S1: Additional characterization of EVs secreted by 19TT CAFs, related to Figure 1. (a) Schematic representation of method used for sEV isolation. 19TT cells were grown in serum-free medium (SFM) and supplemented ± recombinant human (rh)TGF-β1. Obtained conditioned medium (CM) was collected after 2h starvation in SFM. Ultrafiltration (100K NMWL tubes) and differential ultracentrifugation were combined to isolate EV-enriched solution. **(b-e)** sEVs secreted by 19TT CAFs treated ± 5 ng/mL rhTGF-β1 for 2h were

compared. **(b)** BCA assay was used to quantify the total protein content in 19TT-sEV protein extracts. **(c)** Nanoparticle tracking analysis (NTA) was used to determine particle concentration in solutions enriched in 19TT-sEVs. **(d)** TGF-β activity in 19TT-sEVs normalized per μg sEV protein. **(e)** The expression of the extracellular vesicle marker CD63 and TGF-β signaling components was assessed by western blot in 19TT whole cell lysate (WCL) and 19TT-sEV protein extracts. Twenty micrograms of protein extracts were loaded to each lane. **(f)** Migration of MDA231 cells was analyzed by wound healing assay in cell cultures treated ± sEVs secreted by 19TT CAFs treated (TGF-β^{+}) or not treated (TGF-β^{-}) with rhTGF-β1 for 2h. MDA231 cells were treated ± 19TT-sEVs (10 μg/well total protein). **(g)** BMP-SMAD1/5 signaling reporter (Ad-Bre-Fluc) activity, **(h)** IL6/STAT3 signaling reporter (Ad-APRE-Fluc) activity, and **(i)** Wnt/TCF signaling reporter (Ad-TCF-Fluc) activity were quantified in MDA231 cells treated ± sEV TGF-β^{+} secreted by 19TT CAFs (1 μg/well total protein). Recombinant human (rh)IL-6, WNT conditioned medium, and rhBMP4 treatment were used as positive controls. Results represent mean ± SD of at least three independent experiments ($n \geq 3$). Unpaired Student's t-test was used to analyze data in **(b-d)**. One-way ANOVA test followed by Dunn's Multiple Comparison test were used to analyze data in **(e-h).** ns: statistically non-significant, $*p < 0.05$, $**p < 0.01$, $***p < 0.001$.

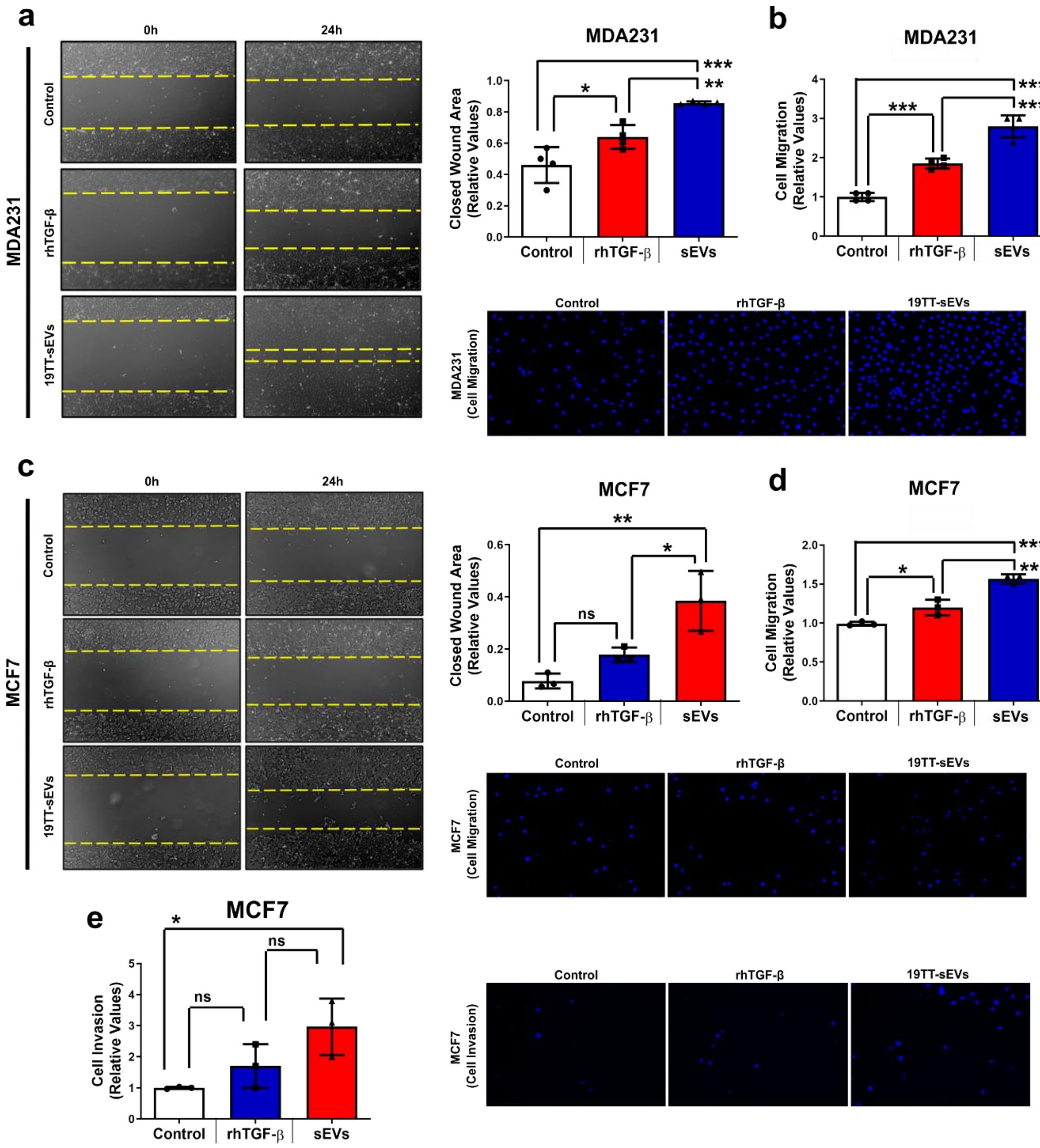

Figure S2: Expanded assessment of cancer cell migration and invasion in response to 19TT-sEVs, related to Figure 4. (a&c) Wound healing assay for **(a)** MDA231 and **(c)** MCF7 cells treated with recombinant human (rh)TGF-β1 or 19TT-sEVs. **(b&d)** Cell migration in transwell inserts. **(b)** MDA231 and **(d)** MCF7 cell migration in transwell inserts (10x magnification). **(e)** MCF7 cell invasion in Matrigel-coated transwell inserts (10x magnification). The concentration of 19TT-sEVs used to treat breast cancer cells was equivalent to 5 ng/mL TGF-β activity. Results represent mean ± SD of at least three independent experiments (n≥3). One-way ANOVA test followed by Dunn's Multiple

Comparison test were used to analyze obtained data. ns: statistically non-significant, *$p < 0.05$, **$p < 0.01$, ***$p < 0.001$.

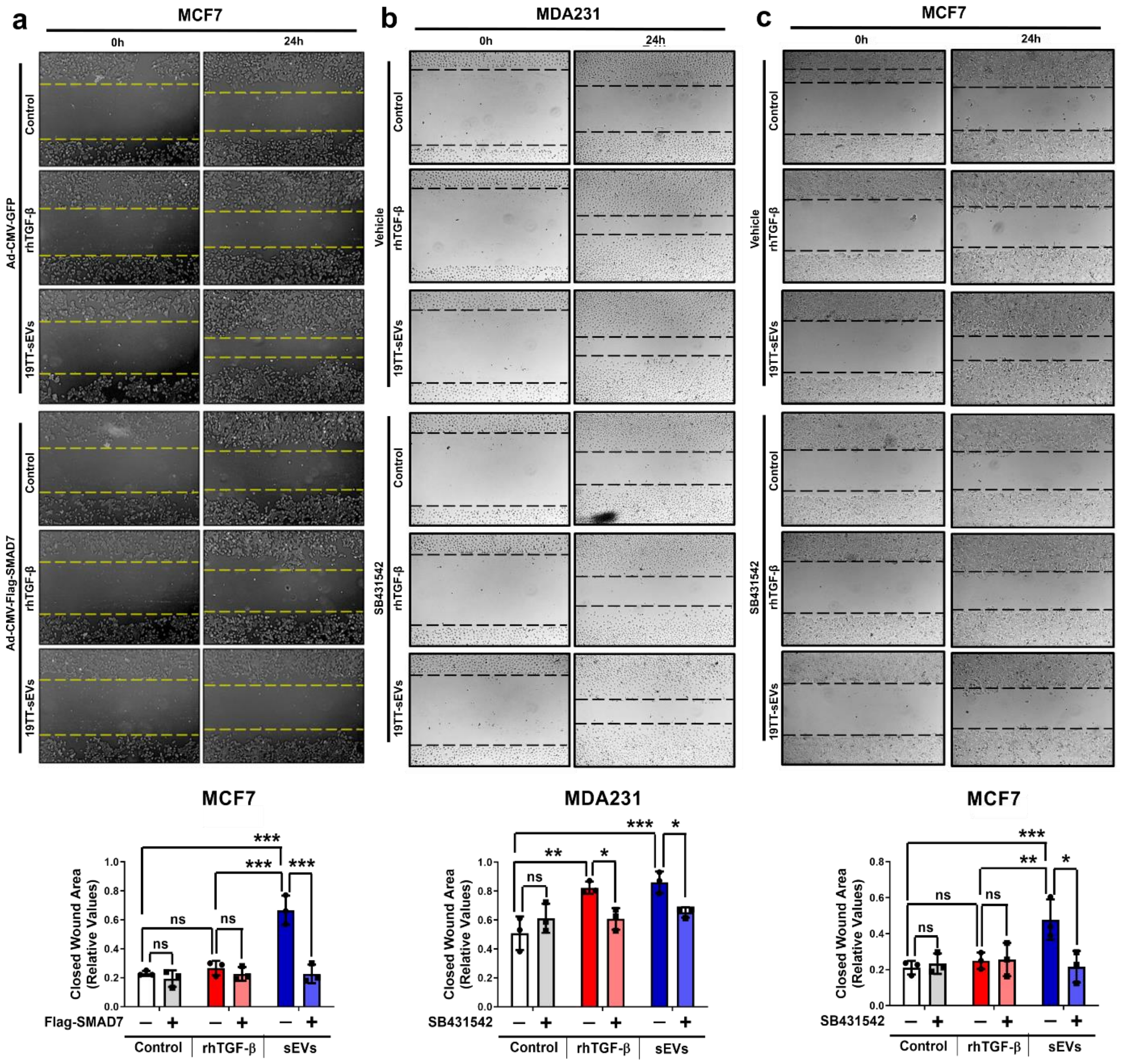

Figure S3: Expanded assessment of cancer cell migration in cell cultures challenged with TGF-β signaling inhibitors, related to Figure 4. (a) Wound healing assay for MCF7 cells infected with Ad-CMV-GFP (control adenovirus) or Ad-CMV-Flag-SMAD7 and treated as indicated. **(b-c)** Wound healing assay for **(b)** MDA231 and **(c)** MCF7 cells treated with vehicle (DMSO) or SB431542. The concentration of 19TT-sEVs used to treat breast cancer cells was equivalent to 5 ng/mL TGF-β activity. Results represent mean ± SD of at least three independent experiments (n≥3). One-way ANOVA test followed by Dunn's Multiple Comparison test were used to analyze obtained data. ns: statistically non-significant, *p < 0.05, **p < 0.01, ***p < 0.001.

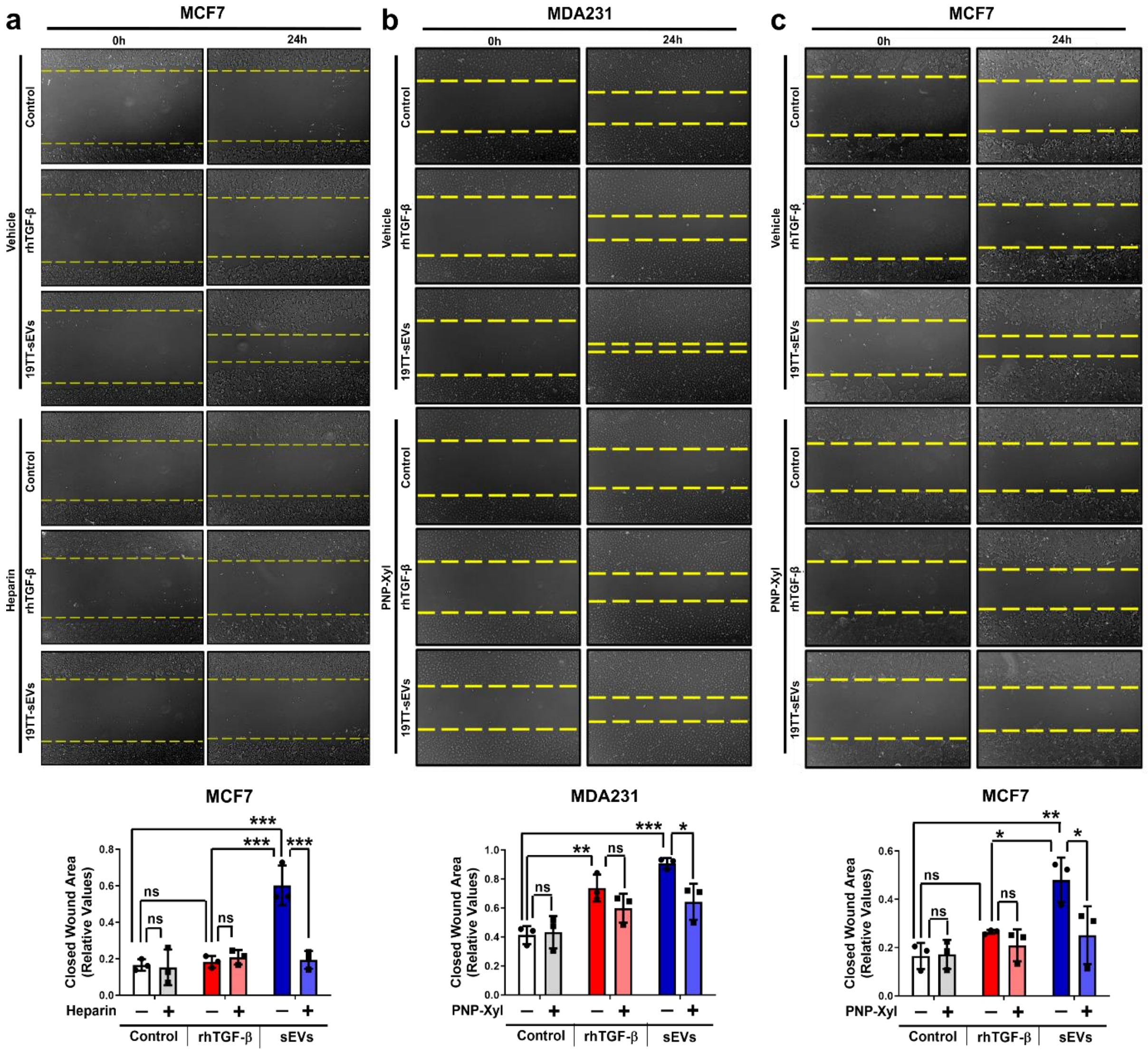

Figure S4: Expanded assessment of cancer cell migration in cell cultures treated with Heparin or PNP-Xyl, related to Figure 4. (a) Wound healing assay for MCF7 cells treated with vehicle or Heparin. **(b-c)** Wound healing assay for **(b)** MDA231 and **(c)** MCF7 cells treated with vehicle (DMSO) or PNP-Xyl. The concentration of 19TT-sEVs used to treat breast cancer cells was equivalent to 5 ng/mL TGF-β activity. Results represent mean ± SD of at least three independent experiments (n≥3). One-way ANOVA test followed by Dunn's Multiple Comparison test were used to analyze obtained data. ns: statistically non-significant, *p < 0.05, **p < 0.01, ***p < 0.001.

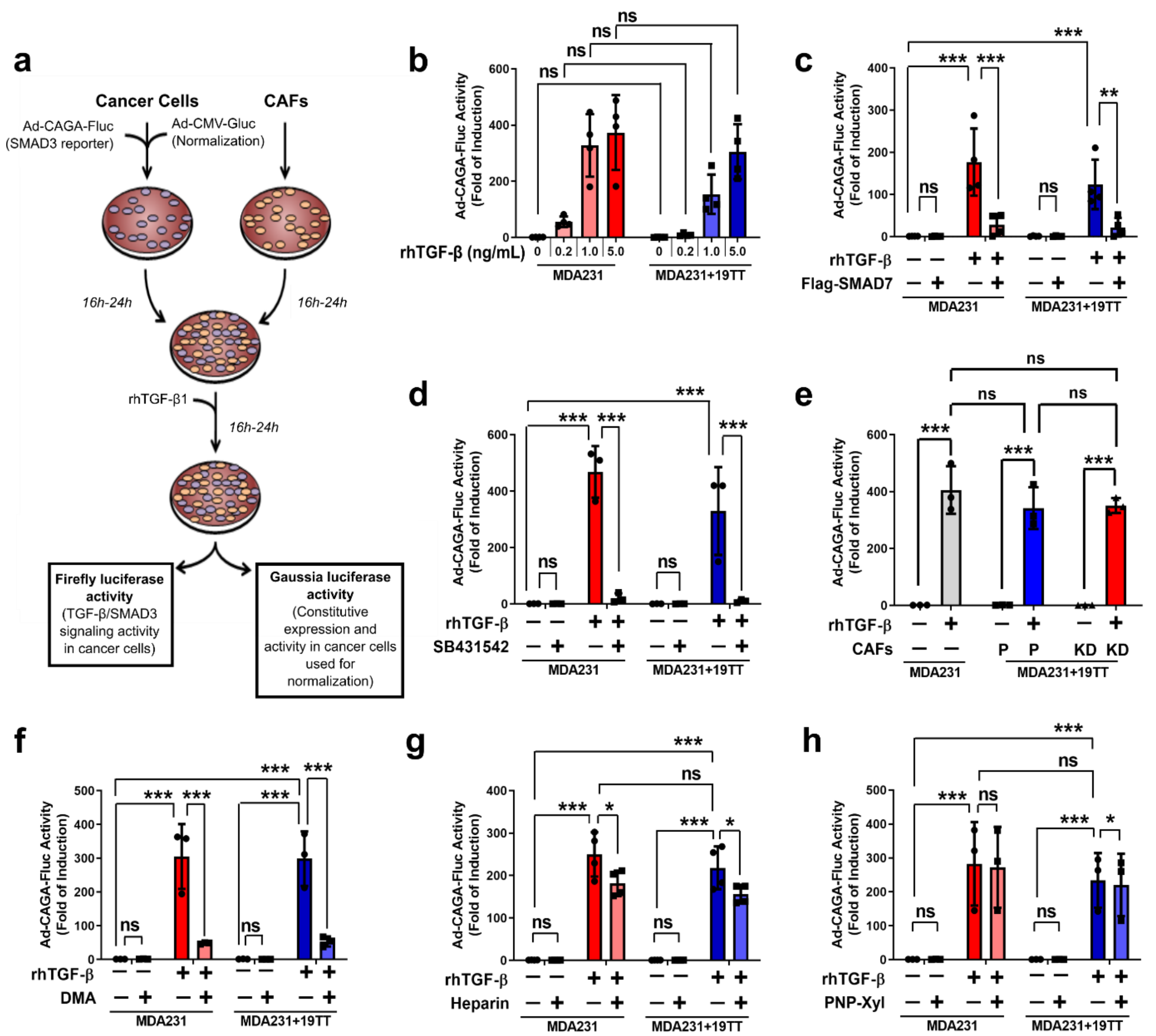

Figure S5: TGF-β signaling activity in MDA231 cells is not significantly impacted by co-culture with 19TT CAFs, related to Figure 5. (a) Schematic illustration of luciferase assay for the quantification of TGF-β/SMAD signaling pathway activity in cancer cells co-cultured with CAFs. **(b-h)** TGF/SMAD3 signaling reporter (Ad-CAGA-Fluc) activity was quantified in MDA231 cells co-cultured ± 19TT CAFs. **(b)** Cell cultures were treated ± recombinant human (rh)TGF-β1 at increasing concentrations for 24h. **(c)** SMAD7 overexpression in MDA231 cells was achieved by infection with Ad-CMV-Flag-SMAD7 and Ad-CMV-GFP was used as control virus. **(d)** Cell cultures were treated ± 2 µM SB431542. **(e)** MDA231 cells were co-cultured ± parental (P) or Rab27a knockdown (KD) 19TT CAFs. Cell cultures were challenged with **(f)** DMA, **(g)** heparin, or **(h)** PNP-Xyl. DMSO was used as a vehicle for SB431542, DMA

and PNP-Xyl. Results represent mean ± SD of at least three independent experiments (n≥3). One-way ANOVA test followed by Dunn's Multiple Comparison test were used to analyze obtained data. ns: statistically non-significant, $**p < 0.01$, $***p < 0.001$.

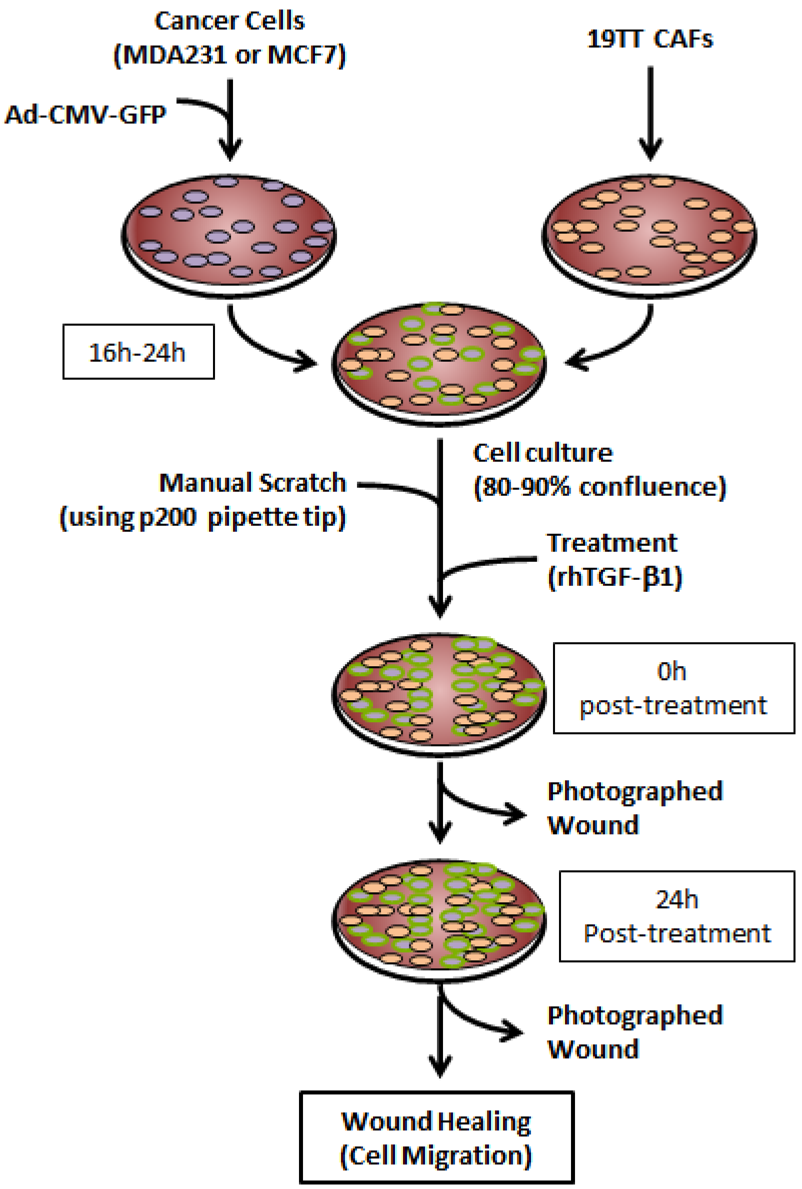

Figure S6: Wound healing assay in co-cultures, related to Figure 6: Schematic illustration of wound healing assay using GFP-labeled cancer cells co-cultured ± 19TT CAFs.

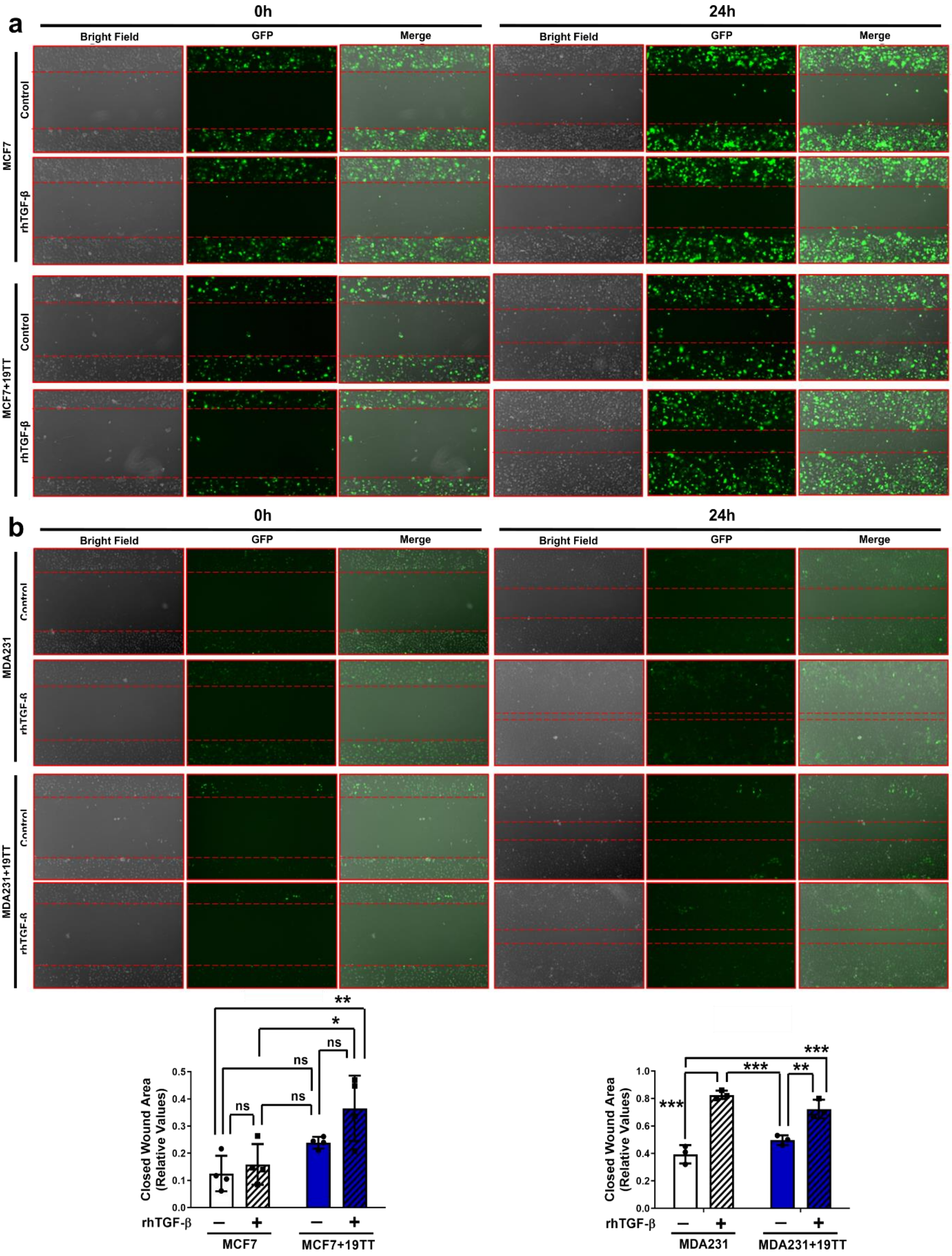

Figure S7: Expanded characterization of CAF-induced breast cancer cell migration, related to Figure 6. Wound healing assay for the assessment of GFP-labeled **(a)** MCF7 or **(b)** MDA231 cell migration in co-cultures ± 19TT cells. Cell cultures were treated ± recombinant

human (rh)TGF-β1. Results represent mean ± SD of at least three independent experiments ($n \geq 3$). One-way ANOVA test followed by Dunn's Multiple Comparison test were used to analyze obtained data. ns: statistically non-significant, $^{**}p < 0.01$, $^{**}p < 0.01$.

103

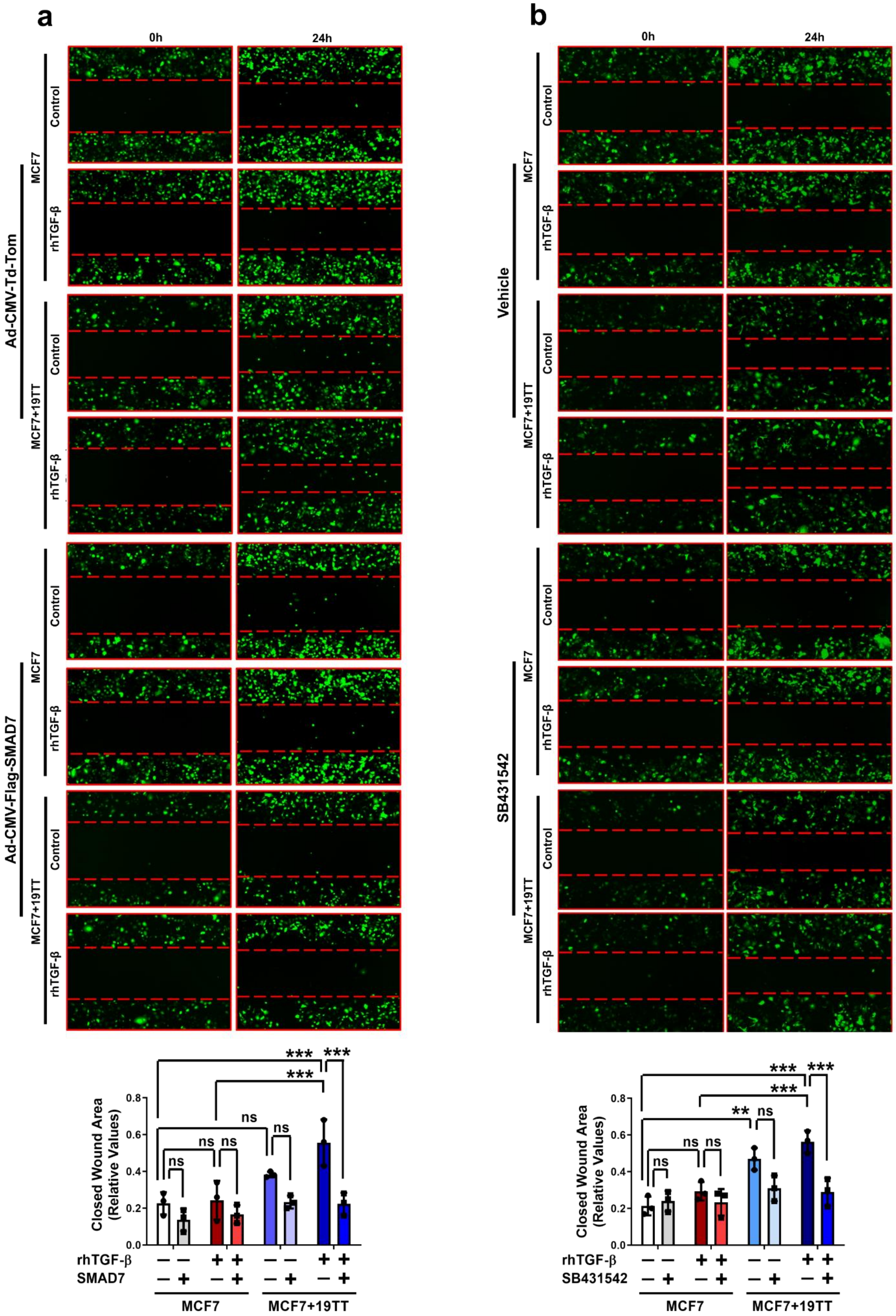
a
0h
24h
Ad-CMV-Td-Tom
Ad-CMV-Flag-SMAD7
MCF7
MCF7+19TT
Control
rhTGF-β
Closed Wound Area
(Relative Values)
rhTGF-β
SMAD7
MCF7
MCF7+19TT
b
Vehicle
SB431542
SB431542

Figure S8: Expanded assessment of CAF-induced breast cancer cell migration in cell cultures challenged with TGF-β signaling inhibitors, related to Figure 6. Wound healing assay for the migration of GFP-labeled MCF7 cells cultured ± 19TT cells and treated ± recombinant human (rh)TGF-β1. **(a)** MCF7 cells were infected with Ad-CMV-Flag-SMAD7 or **(b)** treated with SB431542. Ad-CMV-TdTom was used as control adenovirus. DMSO was used as vehicle for SB431542. Results represent mean ± SD of at least three independent experiments (n≥3). One-way ANOVA test followed by Dunn's Multiple Comparison test were used to analyze obtained data. ns: statistically non-significant, **p < 0.01, **p < 0.01.

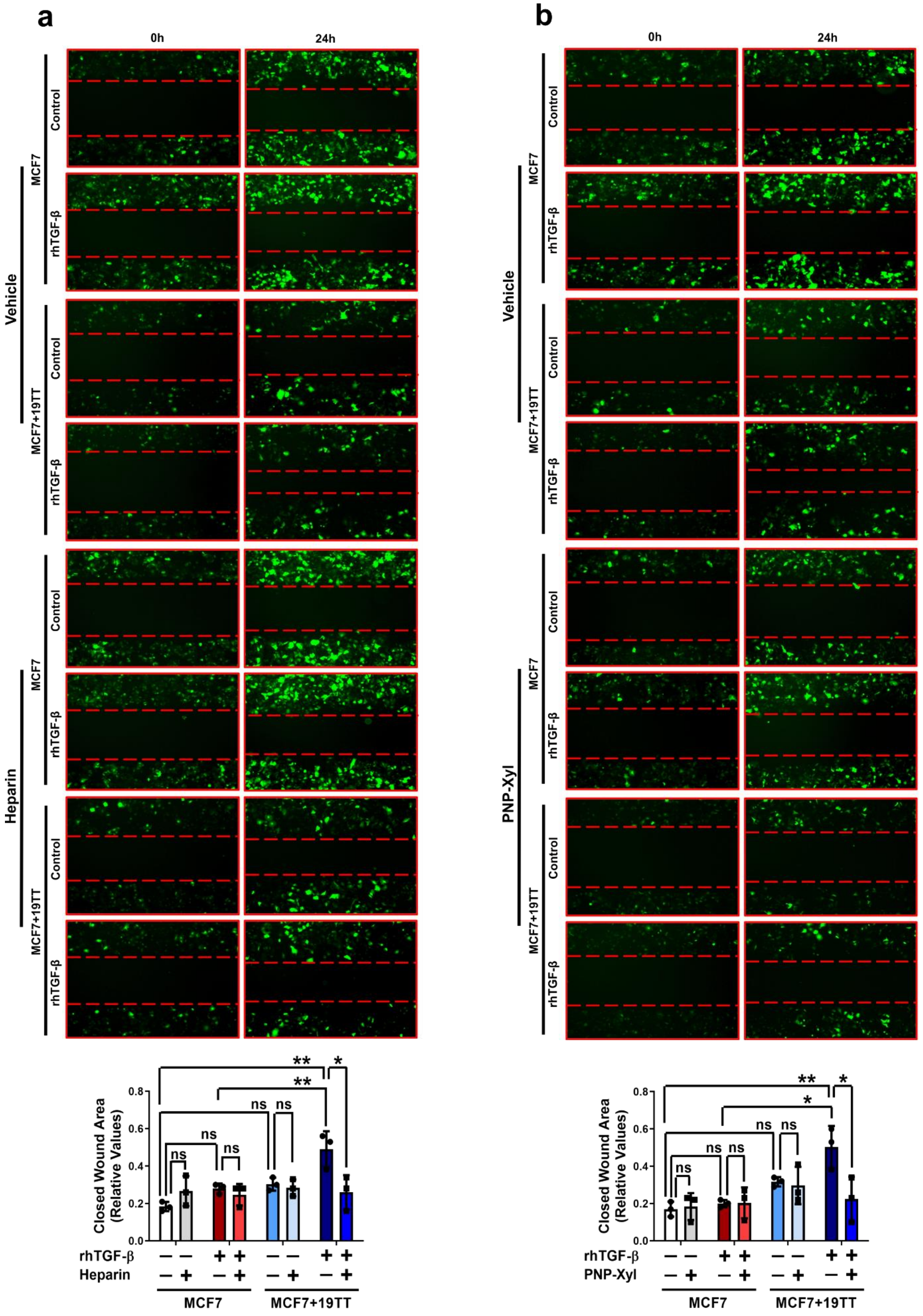
a
b
0h
24h
Control
rhTGF-β
MCF7
MCF7+19TT
Vehicle
Heparin
PNP-Xyl
Closed Wound Area
(Relative Values)
0.0
0.2
0.4
0.6
0.8
ns
**
*
rhTGF-β
Heparin
PNP-Xyl
MCF7
MCF7+19TT

Figure S9: Expanded quantification of CAF-induced breast cancer cell migration in cell cultures treated with Heparin or PNP-Xyl, related to Figure 6. Wound healing assay for the migration of GFP-labeled MCF7 cells cultured ± 19TT cells and treated ± recombinant human (rh)TGF-β1. Cell cultures were treated with **(a)** Heparin or **(b)** PNP-Xyl. DMSO was used as vehicle for PNP-Xyl. Results represent mean ± SD of at least three independent experiments ($n \geq 3$). One-way ANOVA test followed by Dunn's Multiple Comparison test were used to analyze obtained data. ns: statistically non-significant, **$p < 0.01$, **$p < 0.01$.

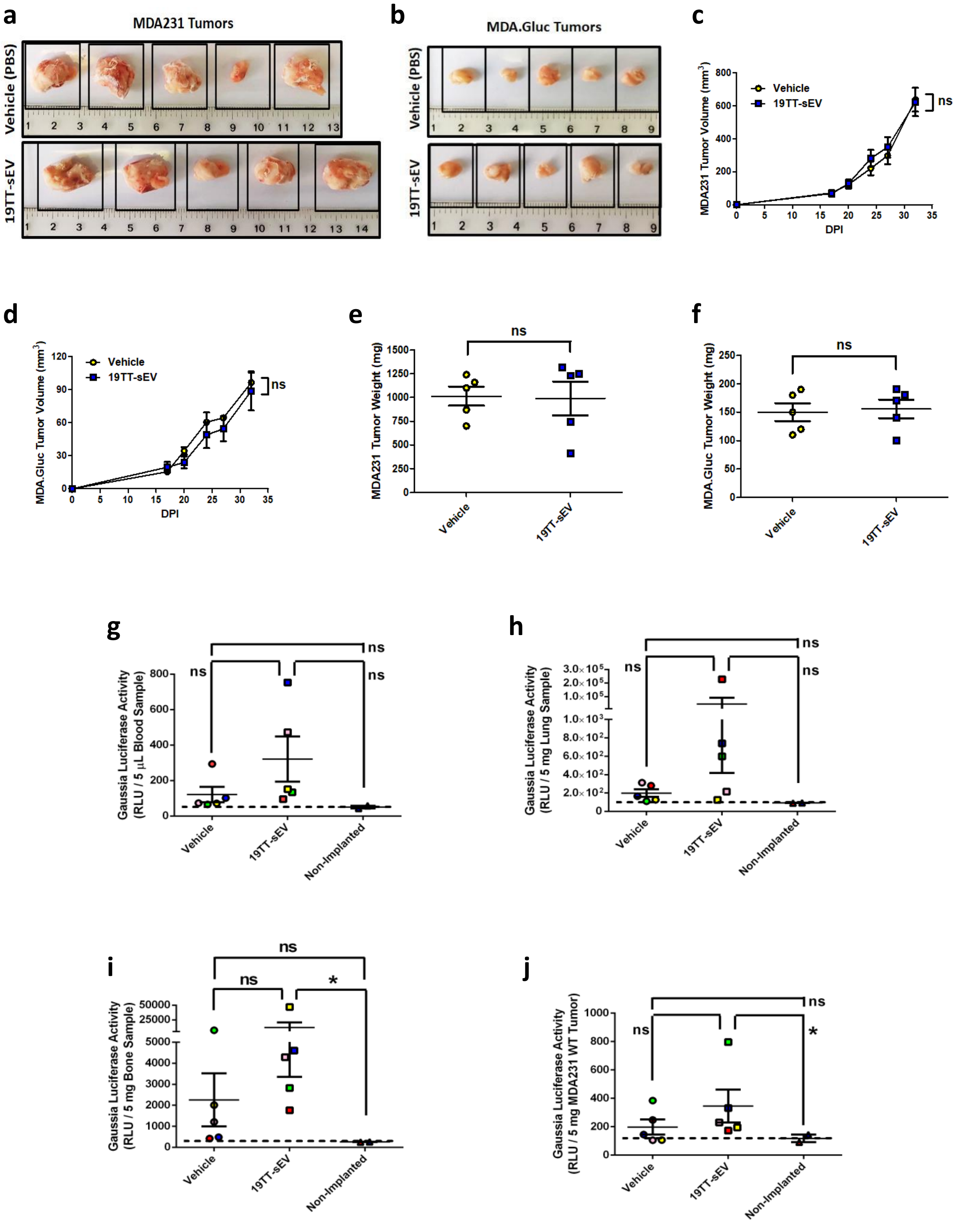
a
MDA231 Tumors
Vehicle (PBS)
19TT-sEV
b
MDA.Gluc Tumors
Vehicle (PBS)
19TT-sEV
c
MDA231 Tumor Volume (mm3)
Vehicle
19TT-sEV
ns
DPI
d
MDA.Gluc Tumor Volume (mm3)
Vehicle
19TT-sEV
ns
DPI
e
MDA231 Tumor Weight (mg)
ns
Vehicle
19TT-sEV
f
MDA.Gluc Tumor Weight (mg)
ns
Vehicle
19TT-sEV
g
Gaussia Luciferase Activity (RLU / 5 μL Blood Sample)
ns
Vehicle
19TT-sEV
Non-Implanted
h
Gaussia Luciferase Activity (RLU / 5 mg Lung Sample)
ns
Vehicle
19TT-sEV
Non-Implanted
i
Gaussia Luciferase Activity (RLU / 5 mg Bone Sample)
ns
*
Vehicle
19TT-sEV
Non-Implanted
j
Gaussia Luciferase Activity (RLU / 5 mg MDA231 WT Tumor)
ns
*
Vehicle
19TT-sEV
Non-Implanted

Figure S10: Additional parameters analyzed for the progression of MDA231 and MDA.Gluc tumors in NOD-SCID mice treated with 19TT-sEVs, related to Figure 7. (a) MDA231 and **(b)** MDA.Gluc tumors collected from animals injected with 19TT-sEVs (or vehicle). **(c)** MDA231 and **(d)** MDA.Gluc tumor volumes were monitored by caliper measurements. **(e)** MDA231 and **(f)** MDA.Gluc tumor weights after harvesting. **(g-j)** Gaussia luciferase activity quantified *ex vivo* in **(g)** blood, **(h)** lung, **(i)** bone, and **(j)** unlabeled MDA231 wild type tumor samples (5 animals/group). Each color represents a sample from a different animal in a particular group. Samples from non-implanted mice (n=2) were used to establish the background activity for the Gaussia luciferase that is represented by a black dashed line. Tissues (organs) were subsampled, and five randomly selected fragments were analyzed by *ex vivo* luciferase assay. Graphs show the average Gaussia luciferase activity calculated for the fragments harvested from each animal. Results represent mean ± SEM. Unpaired Student's t-test was used to analyze data in **(c-f)**. One-Way ANOVA followed by Tukey's Multiple Comparison Test was used to analyze data in **(g-j)**. *p<0.05, **p<0.01, ***p<0.001, ns: statistically non-significant.

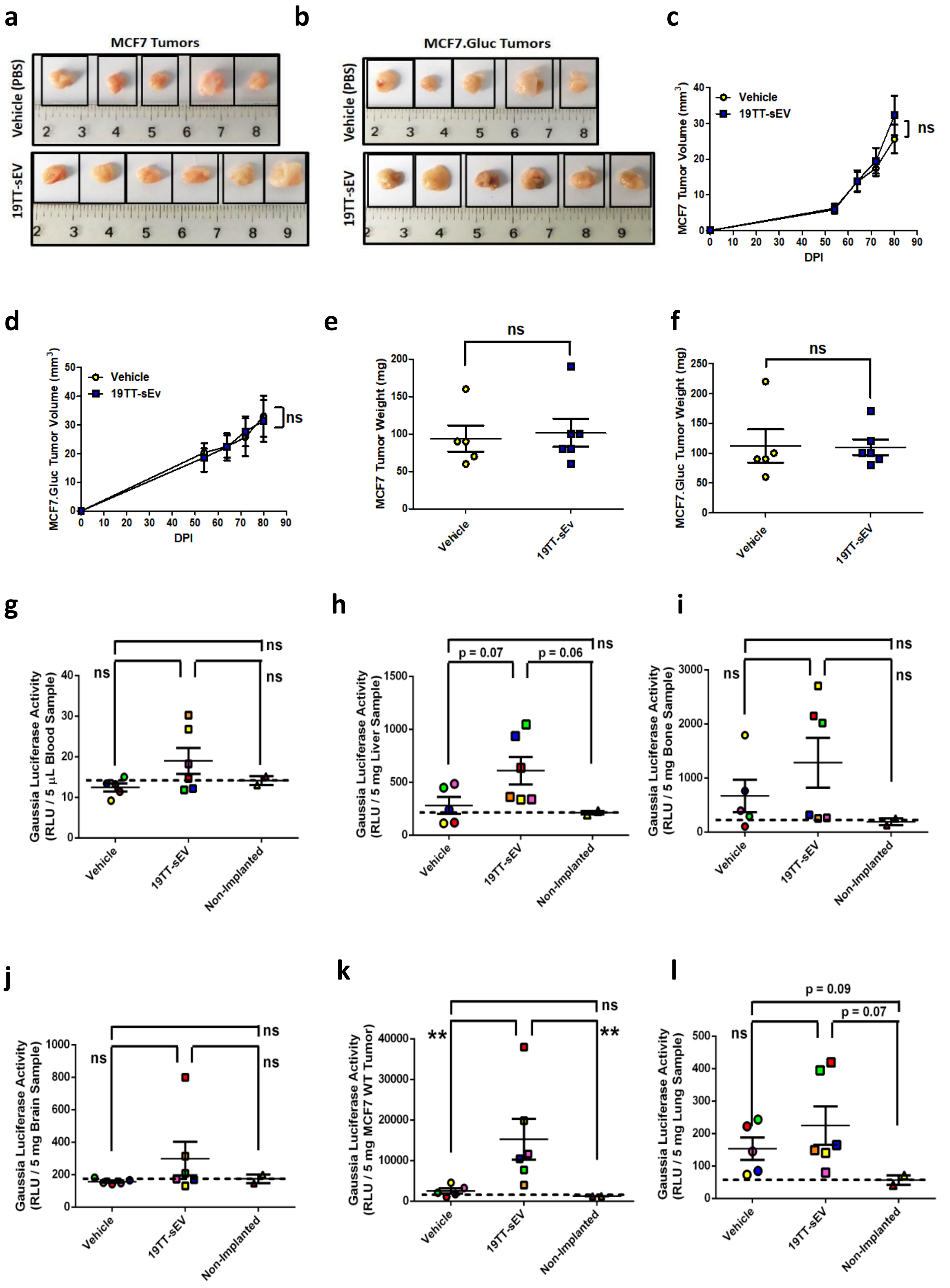
a
MCF7 Tumors
Vehicle (PBS)
19TT-sEV
b
MCF7.Gluc Tumors
Vehicle (PBS)
19TT-sEV
c
Vehicle
19TT-sEV
ns
MCF7 Tumor Volume (mm^3)
DPI
d
Vehicle
19TT-sEv
ns
MCF7.Gluc Tumor Volume (mm^3)
DPI
e
ns
MCF7 Tumor Weight (mg)
Vehicle
19TT-sEv
f
ns
MCF7.Gluc Tumor Weight (mg)
Vehicle
19TT-sEV
g
ns
Gaussia Luciferase Activity (RLU / 5 μL Blood Sample)
Vehicle
19TT-sEV
Non-Implanted
h
p = 0.07
p = 0.06
ns
Gaussia Luciferase Activity (RLU / 5 mg Liver Sample)
i
ns
Gaussia Luciferase Activity (RLU / 5 mg Bone Sample)
j
ns
Gaussia Luciferase Activity (RLU / 5 mg Brain Sample)
k
**
ns
Gaussia Luciferase Activity (RLU / 5 mg MCF7 WT Tumor)
l
p = 0.09
p = 0.07
ns
Gaussia Luciferase Activity (RLU / 5 mg Lung Sample)

Figure S11: Additional parameters analyzed for the progression of MCF7 and MCF7.Gluc tumors in NOD-SCID mice treated with 19TT-sEVs, related to Figure 8. Pictures showing **(a)** MCF7 and **(b)** MCF7.Gluc tumors collected after euthanasia from NOD-SCID mice injected with 19TT-sEVs (or vehicle). **(c)** MCF7 and **(d)** MCF7.Gluc tumor growth as monitored by caliper measurements. **(e)** MCF7 and **(f)** MCF7.Gluc tumor weights. **(g-l)** *Ex vivo* quantification of Gaussia luciferase activity representing the presence of MCF7.Gluc cells assay in **(g)** blood, **(h)** liver, **(i)** bone, **(j)** brain, **(k)** unlabeled MCF7 wild type tumors, and **(l)** lung samples (5-6 animals/group). Colors represent samples harvested from different mice. Background activity for the Gaussia luciferase was quantified in samples from non-implanted mice (n=2) and is represented by black dashed lines. Tissues (organs) were subsampled, and five randomly selected fragments were analyzed by *ex vivo* luciferase assay. Graphs show the average Gaussia luciferase activity calculated for the fragments harvested from each animal. Results represent mean ± SEM. Unpaired Student's t-test was used to analyze data in **(c-f)**. One-Way ANOVA followed by Tukey's Multiple Comparison Test was used to analyze samples in **(g-l)** *p<0.05, **p<0.01, ***p<0.001, ns: statistically non-significant.

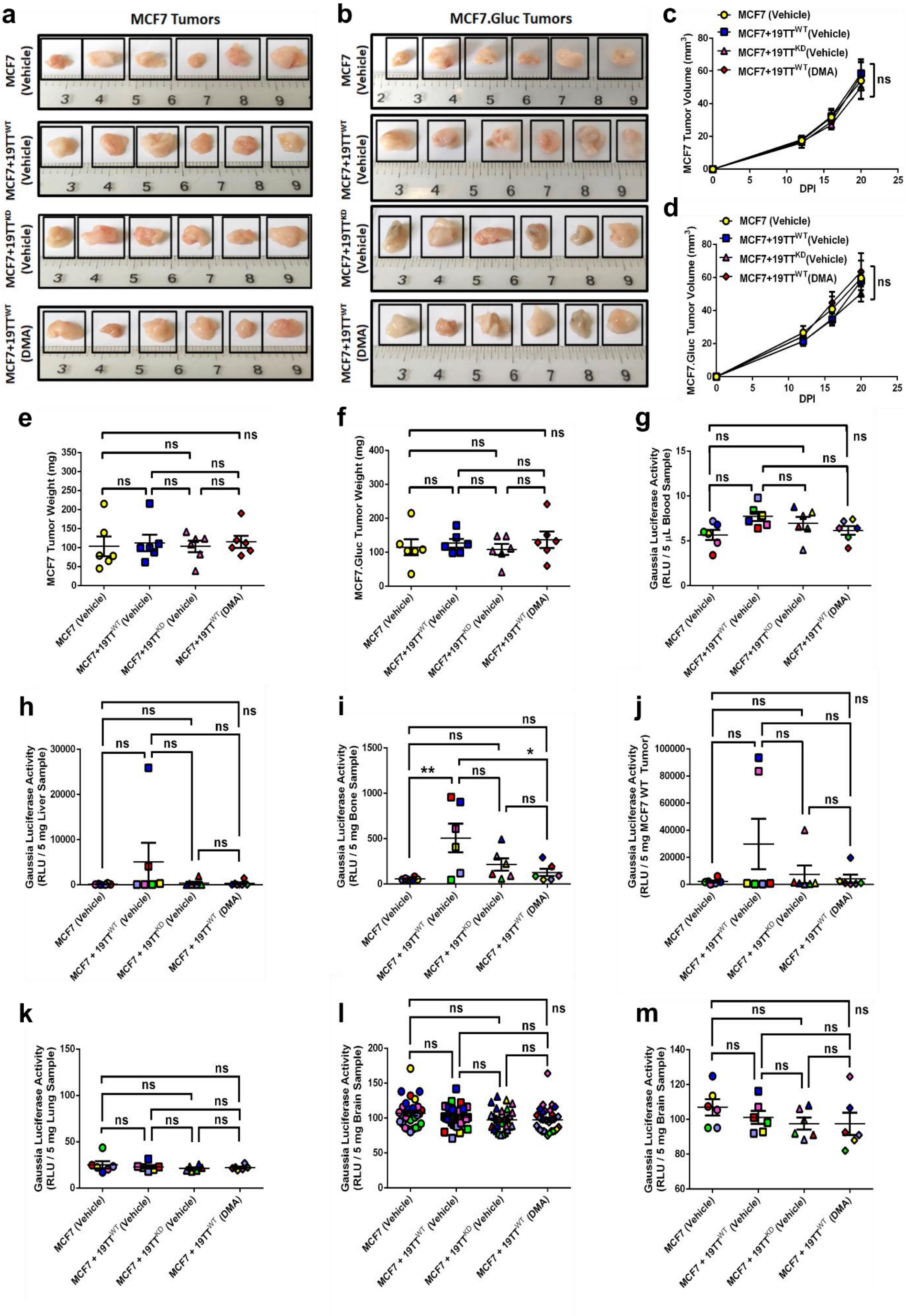
a
MCF7 Tumors
MCF7 (Vehicle)
MCF7+19TT^WT (Vehicle)
MCF7+19TT^KD (Vehicle)
MCF7+19TT^WT (DMA)
b
MCF7.Gluc Tumors
c
MCF7 (Vehicle)
MCF7+19TT^WT(Vehicle)
MCF7+19TT^KD(Vehicle)
MCF7+19TT^WT(DMA)
MCF7 Tumor Volume (mm^3)
DPI
ns
d
MCF7.Gluc Tumor Volume (mm^3)
e
MCF7 Tumor Weight (mg)
f
MCF7.Gluc Tumor Weight (mg)
g
Gaussia Luciferase Activity (RLU / 5 μL Blood Sample)
h
Gaussia Luciferase Activity (RLU / 5 mg Liver Sample)
i
Gaussia Luciferase Activity (RLU / 5 mg Bone Sample)
**
*
j
Gaussia Luciferase Activity (RLU / 5 mg MCF7 WT Tumor)
k
Gaussia Luciferase Activity (RLU / 5 mg Lung Sample)
l
Gaussia Luciferase Activity (RLU / 5 mg Brain Sample)
m
Gaussia Luciferase Activity (RLU / 5 mg Brain Sample)
MCF7 + 19TT^WT (Vehicle)
MCF7 + 19TT^KD (Vehicle)
MCF7 + 19TT^WT (DMA)

Figure S12: Expanded characterization of impacts caused by impaired EV trafficking on CAF-induced breast cancer progression, related to Figure 9. (a) MCF7 and **(b)** MCF7.Gluc (± 19TT) tumors harvested from NOD-SCID mice after euthanasia. Growth of **(c)** MCF7 and **(d)** MCF7.Gluc (± 19TT) tumors was monitored by caliper measurements. **(e)** MCF7 and **(f)** MCF7.Gluc (± 19TT) tumor weights. **(g-m)** Gaussia luciferase activity quantified by *ex vivo* luciferase assay in **(g)** blood, **(h)** liver, **(i)** bone, **(j)** unlabeled MCF7 wild type tumors, **(k)** lung, and **(l-m)** brain samples (6 animals/group). Mice are color-coded. Tissues (organs) were fractionated, and five subsamples were randomly chosen for further analysis. Average Gaussia luciferase activity is shown for each animal in **(g-k & m)**. Absolute Gaussia luciferase activity is shown for individual samples (5 samples/mouse) in **(l)**. Results represent mean ± SEM. One-Way ANOVA followed by Tukey's Multiple Comparison Test was used to analyze samples in **(c-m)** *$p<0.05$, **$p<0.01$, ***$p<0.001$, ns: statistically non-significant.
